# Preeclampsia prediction and diagnosis: a comprehensive historical review from clinical insights to omics perspectives

**DOI:** 10.3389/fmed.2025.1689745

**Published:** 2025-10-23

**Authors:** Julian C. Riano-Moreno, Elizabeth Vargas-Castellanos, Amy Pedraza, Laura Sofía Díaz-Quiñonez, Víctor Saul Rangel-Ramos

**Affiliations:** ^1^Medical Subdirection, National Institute of Cancerology, Bogotá, DC, Colombia; ^2^Faculty of Medicine, Cooperative University of Colombia, Villavicencio, Colombia; ^3^Bioethics Department, Universidad El Bosque, Bogotá, DC, Colombia; ^4^Hospital Universitario Mayor-Méderi, Universidad del Rosario, Bogotá, DC, Colombia; ^5^Universidad del Rosario, Bogotá, DC, Colombia

**Keywords:** preeclampsia, biomarkers, omics technologies, hypertensive disorders, history, artificial intelligence

## Abstract

Preeclampsia (PE) is a multifactorial and multisystemic syndrome specific to human pregnancy, traditionally characterized by hypertension and proteinuria. Affecting 2%−10% of pregnancies, PE remains a leading cause of maternal and perinatal morbidity and mortality, particularly in low- and middle-income countries. This review traces the historical evolution of PE diagnosis, from early clinical observations to the incorporation of modern omics biomarkers. Early diagnostic criteria were based on observable clinical symptoms, but advancements in biomedical science have highlighted the significance of angiogenic and anti-angiogenic factors, such as sFlt-1 and PlGF, which are used as “Established Angiogenic Biomarkers.” Despite these advancements, clinical consensus on the use of these biomarkers remains elusive due to their variable sensitivity. Recent integrative approaches using omics technologies have provided deeper insights into the complex pathophysiology of PE, uncovering new pathways and “Potential Molecular Biomarkers” for early diagnosis and prediction. However, challenges remain in translating these findings into clinical practice, particularly due to the need for robust validation studies and the consideration of inter-individual and population variability. This review emphasizes the importance of continued research and validation of these biomarkers in diverse cohorts to develop effective predictive tools and improve maternal and fetal outcomes. By exploring the historical and modern perspectives on PE diagnosis, this review aims to provide a comprehensive understanding of the disease and highlight future directions in PE research.

## 1 Introduction

Preeclampsia (PE) is a multifactorial and complex multiorgan syndrome specific to human pregnancy and the puerperium. Traditionally, it has been characterized by the presence of hypertension with evidence of end-organ involvement, such as proteinuria, thrombocytopenia, renal insufficiency, pulmonary edema, or cerebral symptoms after 20 weeks' gestation (1, 2). Preeclampsia complicates 2%−10% of all pregnancies, and more than 99% of the annual 46,000 preeclampsia-related maternal deaths occur in low-income and middle-income countries (3). Although it is a well-recognized disease of pregnancy, it has historically been poorly defined due to changing diagnostic criteria over the years (4).

The evolving technoscientific and biomedical understanding of its etiology over time has contributed to clarifying the diagnosis and even predicting this condition. However, the etiology remains incompletely understood (4). Several biomedical advances have provided multiple diagnostic and predictive biomarkers for PE, but there is currently no consensus on their clinical use (5, 6).

**Figure 1:**
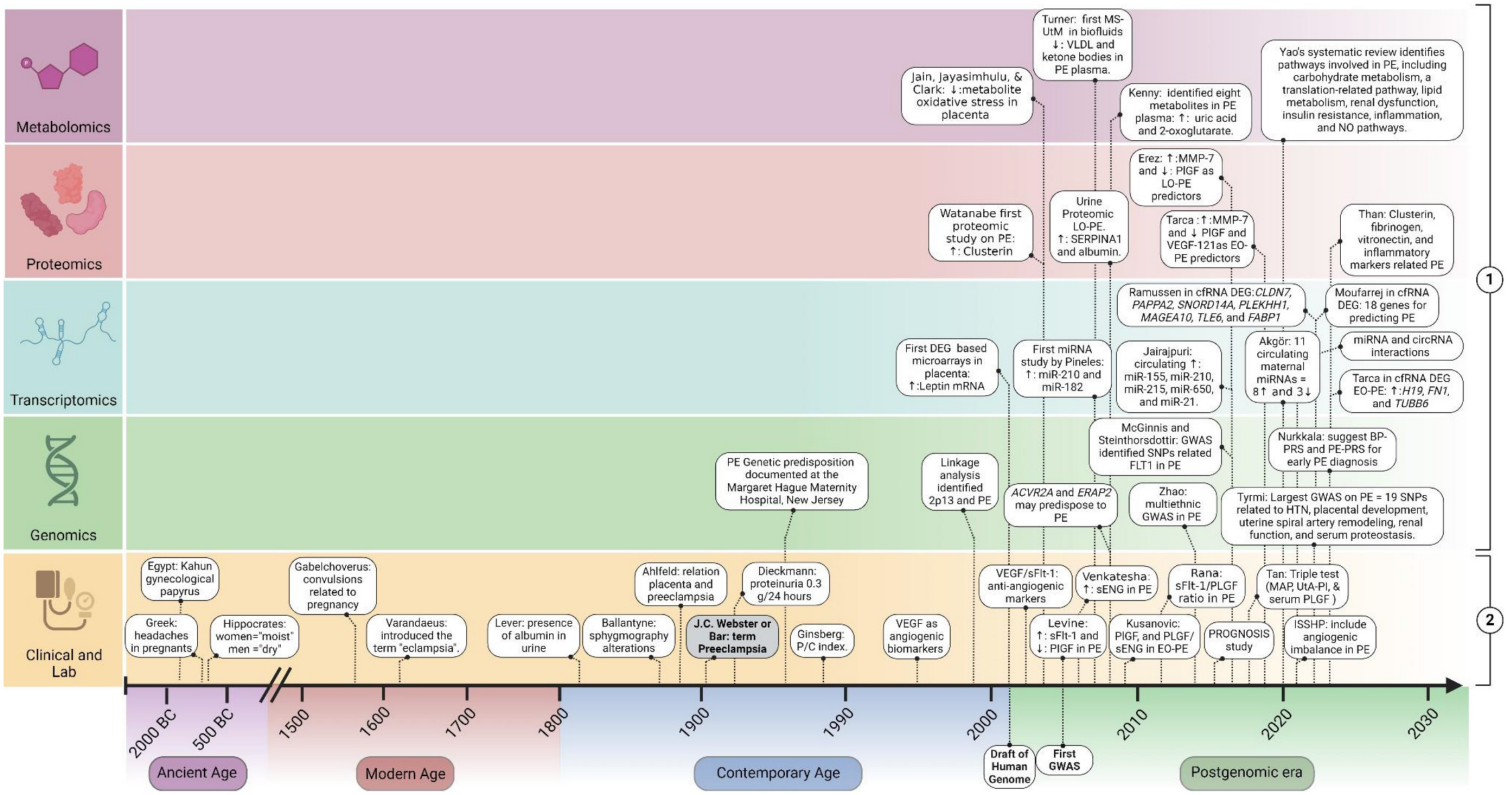
Timeline of milestones in biomarker discovery for prediction and diagnosis of preeclampsia. This figure illustrates the chronological progression of biomarker discovery in the prediction and diagnosis of PE. It includes two main categories: (1) potential Molecular Biomarkers: Omics studies findings to diagnose and predict PE; (2) clinical criteria and “Established Angiogenic Biomarkers” in PE. EO-PE, early-onset PE; LO-PE, late-onset PE; MAP, mean arterial pressure; UtA-PI, uterine artery pulsatility index; DEG, differential expressed genes studies; sENG, soluble endoglin; VEGF, vascular endothelial growth factor; PlGF, placental growth factor; sFlt-1, soluble fms-like tyrosine kinase-1. Created with BioRender.com.

To improve this understanding, we present a historical perspective on the diagnostic evolution of preeclampsia (PE), tracing the development from clinical and laboratory biomarkers to the latest advancements in omics biomarkers across different Eras: Ancient, Modern, Contemporary, and the Postgenomic era. By examining the progression of diagnostic criteria and the incorporation of potential novel biomarkers, we aim to elucidate how these changes have enhanced our ability to understand the underlying biological mechanisms of PE, while also opening new avenues for predicting and diagnosing the condition. This review highlights significant milestones (Figure 1) and future directions in the prediction and diagnosis of preeclampsia.

## 2 Evolution of the diagnosis of hypertensive disorders in pregnancy

Hypertensive disorders of pregnancy (HDP) have been part of medical history since ancient times. The earliest related records date back to Ancient Age in Egypt, specifically the Kahun gynecological papyrus (1850–1700 BC), where treatments were described to prevent the pregnant woman from biting her tongue during childbirth (7). The ancient Greeks also mentioned associated symptoms, such as headaches in pregnant women, which are currently considered a precursor sign of some types of HDP (4, 8).

In the Modern Age, the clinical manifestations of HDP began to be described, starting with convulsions as the first concrete clinical sign associated with these conditions. In 1596, Gabelchoverus included convulsions as a specific group related to pregnancy or originating in the uterus in his work (8). However, it was Johannes Varandaeus who introduced the term “eclampsia” from the Greek “eklampsis,” meaning “a flash,” in his 1620 treatise “Tractatus de affectibus Renum et Vesicae,” which focused on convulsions associated with pregnancy (7, 9). Further, François Mauriceau in 1637 identified convulsions predominantly in primigravida women, proposing primigravidity as a risk factor for eclampsia (4). Additionally, François Boissier de Sauvages, a French physician and botanist, distinguished the convulsive episodes of eclampsia from those of epilepsy (7). This differentiation allowed eclampsia to be recognized in 1793 as a pathology distinct from epileptic processes, definitively integrating it into obstetric pathologies (4).

The laboratory manifestations of HDP were described in the Contemporary Age. In 1843, John C. W. Lever described the presence of albumin in the urine of women with puerperal convulsions, a finding confirmed by Sir James Young Simpson, a pioneer of modern obstetrics. Thus, for the first time, proteinuria was correlated with eclampsia. In 1940, William J. Dieckmann established a proteinuria cutoff of 0.3 g every 24 h for 3 days as abnormal in pregnancy (4). Further, in 1983, Ginsberg et al. de-emphasized proteinuria as the sole marker of eclampsia by introducing the proteinuria/creatininuria (P/C) index, considering 24-h proteinuria as a more suitable index for the diagnosis of eclampsia (10). This, along with studies establishing the relationship between proteinuria and maternal-fetal prognosis, relegated the routine measurement of 24-h proteinuria to a severity criterion (11), while the P/C index is increasingly considered a potential “Gold Standard” for diagnosis (10, 12).

On the other hand, a well-known sign previously considered pathognomonic of eclampsia and proteinuria, edema, described by Demanet in 1797 and supported by the studies of Friedman and Neff in 1977, has been dismissed for its non-specificity (13).

The first established relationship between neurohypertensive or vasospastic symptoms and eclampsia was in 1843 by Robert Johns, who identified these as warning symptoms of eclampsia (8). This relationship was confirmed in 1885 by John William Ballantyne, who observed that pregnant women with edema and albuminuria exhibited alterations in sphygmography reports (7). However, it was not until 1897 that Vasquez and Nobecourt established a clear correlation between eclampsia and gestational hypertension. Since then, arterial hypertension during pregnancy has become a diagnostic criterion for disorders such as PE.

In 1972, the American College of Obstetricians and Gynecologists (ACOG) established and specified week 20 as the cutoff point for the different hypertensive manifestations in pregnancy, primarily considering diastolic blood pressure and defining it as altered when values exceeded 90 mmHg (4, 8). Currently, there is controversy regarding the stages of blood pressure measurement, as the American Heart Association (AHA) has proposed stage cutoffs that focus not only on the second and third trimesters but also on the first trimester and prenatal stages (14, 15).

## 3 Evolution of the concept and clinical diagnosis of preeclampsia

Although eclampsia has traditionally been seen as the main feature of HDP, it is now recognized that hypertension and proteinuria precede convulsions. This preceding state is considered a different clinical condition within the HDP spectrum, called PE. While there is no consensus on who introduced the term, Leon Chesley attributed its introduction to John Clarence Webster in 1903 in the United States and to Bar in France (7). Since this condition is a precursor to eclampsia, most clinical efforts have focused on the diagnosis and prediction of PE to prevent the devastating impacts of eclampsia.

Currently, PE is recognized as a complex and multisystemic disease. Postmortem findings reveal macroscopic damage in the brain, kidneys, liver, hematopoietic system, and fetoplacental unit, and less frequently in the lungs, heart, pancreas, eyes, intestines, and endocrine and immune systems (7, 16). These damages have been associated with global maternal endothelial dysfunction (17). The state of maternal endothelial dysfunction and subsequent multisystem dysfunction has led to multiorgan damage being included as a diagnostic criterion for PE since 2000 (17, 18).

In addition to hypertension, PE is related to other hemodynamic changes associated with the maternal-fetal interaction, exhibiting diverse hemodynamic phenotypes. For instance, fetal growth restriction (FGR) without PE is more related to high peripheral vascular resistance (19). Conversely, when PE and FGR occur together, a distinct cardiovascular phenotype emerges, characterized by decreased cardiac output and increased vascular resistance. This suggests that fetal weight and gestational age are related to differences in the presentation of maternal endothelial dysfunction, highlighting the various ways in which PE can manifest (19, 20).

These variations in PE manifestation have led to the identification of two distinct forms: early-onset PE (EO-PE) and late-onset PE (LO-PE) (21, 22). This classification considers not only the gestational age at onset but also the underlying mechanisms of each form. EO-PE, which occurs before 34 weeks of gestation, is associated with abnormal placentation. This condition is often linked to severe placental insufficiency resulting from inadequate remodeling of the spiral arteries, leading to reduced placental perfusion. EO-PE has a stronger genetic component and is associated with a higher risk of recurrence within families, indicating a significant hereditary influence (23).

In contrast, LO-PE, presenting after 34 weeks, arises from the interaction between a normally developed placenta and a maternal predisposition to PE. This predisposition is often determined by pre-existing microvascular diseases such as hypertension, diabetes, or other metabolic imbalances. Consequently, LO-PE is less about placental abnormalities and more about the maternal body's inability to cope with the increased physiological demands imposed by the growing fetoplacental unit. This failure of the maternal cardiovascular system to adapt to pregnancy demands highlights the complex interplay between maternal health and fetal development (23).

Therefore, PE is not only a maternal multisystem disease but also a maternal-fetal disease, reflecting the intricate relationship between maternal health and fetal development (18). The historical fluctuation and clinical diversity of HDP have created significant difficulties in establishing specific diagnostic criteria for each of these conditions (24). This has led to discrepancies between scientific societies in defining diagnostic criteria for PE (Table 1). Consequently, it has become necessary to resort to additional strategies beyond traditional clinical and paraclinical evaluations to achieve an accurate diagnosis of PE. Although, the etiology of PE remains poorly understood, efforts to understand the underlying mechanisms have enabled the introduction of new methods to predict and diagnose PE.

**Table 1:** Diagnostic criteria for hypertensive pregnancy disorders from various gynecology and obstetrics societies.

**Category**	**Societies of gynecology and obstetrics**				
	**ACOG**	**SOGC**	**RCOG**	**SOMANZ**	**ISSHP**
Gestational hypertension	Increase in blood pressure after 20 weeks of gestation, without accompanying proteinuria	New hypertension at ≥ 20 weeks of gestation	New hypertension after 20 weeks without significant proteinuria	New hypertension after 20 weeks without maternal or fetal PE characteristics followed by normalization within 3 months postpartum	New-onset hypertension after 20 weeks of gestation in the absence of proteinuria and maternal or organ-uteroplacental dysfunction
PE/eclampsia	Hypertension with proteinuria (24 h excretion ≥ 300 mg) after 20 weeks of gestation until 2 weeks postpartum or new HTN with one or more of the following: platelets < 100,000/μl, serum creatinine >1.1 mg/dl, elevated transaminases, pulmonary edema, cerebral/visual symptoms	Gestational hypertension with one or more of the following: new proteinuria, adverse conditions, severe complications	New hypertension with significant proteinuria after 20 weeks or eclampsia as a convulsive condition associated with PE or severe PE with severe HTN and/or biochemical/hematological symptoms	Multisystemic with new HTN after 20 weeks or a multisystem disorder characterized by HTN and involvement of one or more organs or the fetus	New-onset hypertension after 20 weeks of gestation with proteinuria and maternal/uteroplacental organ dysfunction
Superimposed PE/eclampsia on chronic hypertension	Hypertension diagnosed before or in early pregnancy with development of proteinuria	Hypertension with one or more of the following at ≥ 20 weeks: resistant HTN, new or worsened proteinuria, adverse conditions, severe complications	Not specified	Woman with chronic HTN developing one or more systemic features of PE after 20 weeks of gestation	One or more of the following (proteinuria and maternal/uteroplacental organ dysfunction) in addition to HTN

HTN, hypertension.

## 4 Exploring the etiological hypotheses of preeclampsia

Several hypotheses about the etiology of PE have been proposed over time. In the Ancient times, Hippocrates described women as “moist” and men as “dry”, which he believed predisposed women to fluid absorption, leading to edema during pregnancy. Others considered the uterus to be a wandering organ that, in its displacement, damaged other organs (8). Additionally, an infectious hypothesis was proposed, suggesting that PE was caused by the infection of *Bacillus eclampsiae*, now known as *Proteus vulgaris*. François Mauriceau attributed PE to abnormalities in lochial flow or intrauterine fetal death (4, 8).

One of the most recognized hypotheses about the etiology of PE, originating from Modern times, the idea that toxins or poisons entering the maternal bloodstream could produce the symptoms. This hypothesis has given rise to widely used terms in the clinical context, such as “toxemia of pregnancy” or “gravidic toxemia”, which encompass not only PE but also eclampsia, hyperemesis gravidarum, acute yellow atrophy of the liver, *gravidic pruritus*, and *ptyalism* (7). However, although our understanding of the disease has advanced significantly from these early hypotheses, the exact cause of PE remains elusive.

In contrast to these earlier hypotheses, the uteroplacental dysfunction hypothesis has garnered the most support in contemporary research. The initial connection between the placenta and PE was proposed by Ahlfeld in 1894 (4). This hypothesis gained further validation in 1914 when James Young identified a relationship between placental infarctions and the development of eclampsia. Subsequent experimental studies demonstrated that guinea pigs administered placental extracts from PE women developed convulsions and other symptoms associated with eclampsia (7). Positioning placental dysfunction as a central factor in the pathogenesis of PE has paved the way for further research and study of PE.

During the second half of the 20th century, significant research efforts were directed toward understanding the relationship between the placenta and PE. One remarkable observation resulting from these studies was that trophoblast cells in PE failed to adequately invade the maternal spiral arteries and convert them from small muscular vessels into large, low-resistance vessels. This finding suggested an “uteroplacental antiangiogenic state” as the main pathophysiological mechanism of the disease (8), which precedes the maternal endothelial dysfunction associated with the multisystemic nature of PE. This concept was termed the “two-stage theory of PE.” Thus, the study and identification of uteroplacental antiangiogenic factors offer a rational starting point for exploring the pathophysiology of PE (25, 26). These insights were crucial for reevaluating diagnostic and predictive methods for PE.

Currently, it is recognized that neither blood pressure nor proteinuria is specific enough to define PE, and relying solely on these methods can lead to overdiagnosis of the disease (27). Therefore, the inclusion of measurements of factors related to the “uteroplacental antiangiogenic state” alongside traditional methods has become a cornerstone for a more accurate and reliable diagnosis of PE (26, 28). As a result, the International Society for the Study of Hypertension in Pregnancy (ISSHP) proposed a new definition of PE in 2021 that includes uteroplacental and target organ dysfunction, as well as the measurement of antiangiogenic biomarkers in maternal circulation as part of the diagnostic criteria for PE (1).

## 5 Angiogenic and anti-angiogenic factors: key discoveries in preeclampsia

**Figure 2:**
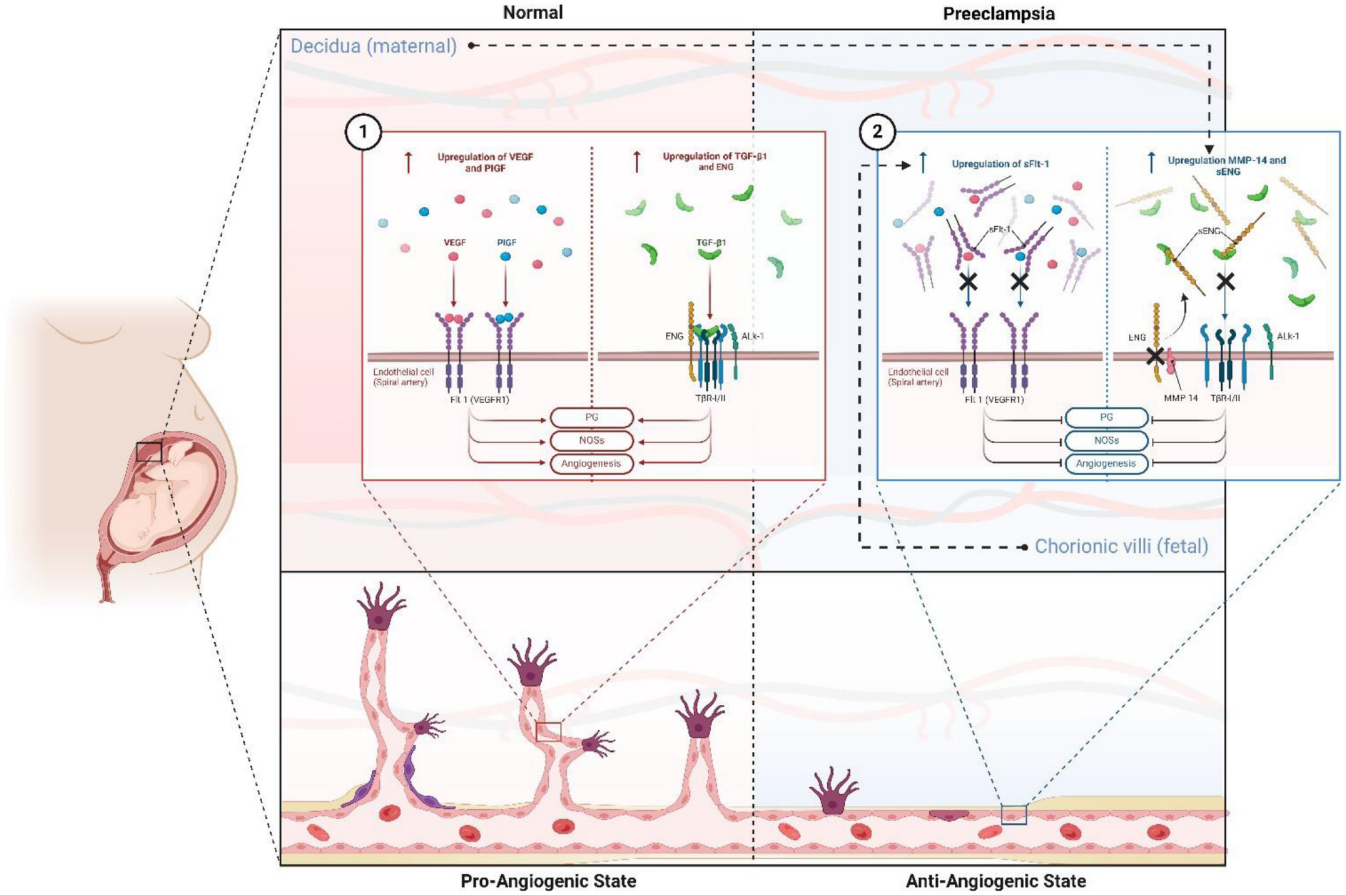
Antiangiogenic and proangiogenic state and factors in normal gestation and preeclampsia. In normal pregnancy (1), there is a pro-angiogenic state mediated by the upregulation and increase of Flt-1 ligands such as PlGF and VEGF, and TNF-β1 pathway activity supported by increased expression of the ENG coreceptor. This favors PG liberation, NOS activity, ON liberation, vasodilation, and angiogenesis. However, in PE, this state is reversed (2), leading to an antiangiogenic state primarily due to fetal-stimulated expression of sFlt-1, which competes for ligands PlGF and VEGF. Additionally, maternal upregulation of MMP-14 increases sENG, which antagonizes the TNF-β1 pathway. ENG, endoglin; sENG, soluble endoglin; Flt-1: fms-related receptor tyrosine kinase 1; VEGF, vascular endothelial growth factor; PlGF, placental growth factor; sFlt-1, soluble fms-like tyrosine kinase-1; NOSs, nitric oxide synthase; ON, nitric oxide. Created with BioRender.com.

**Figure 3:**
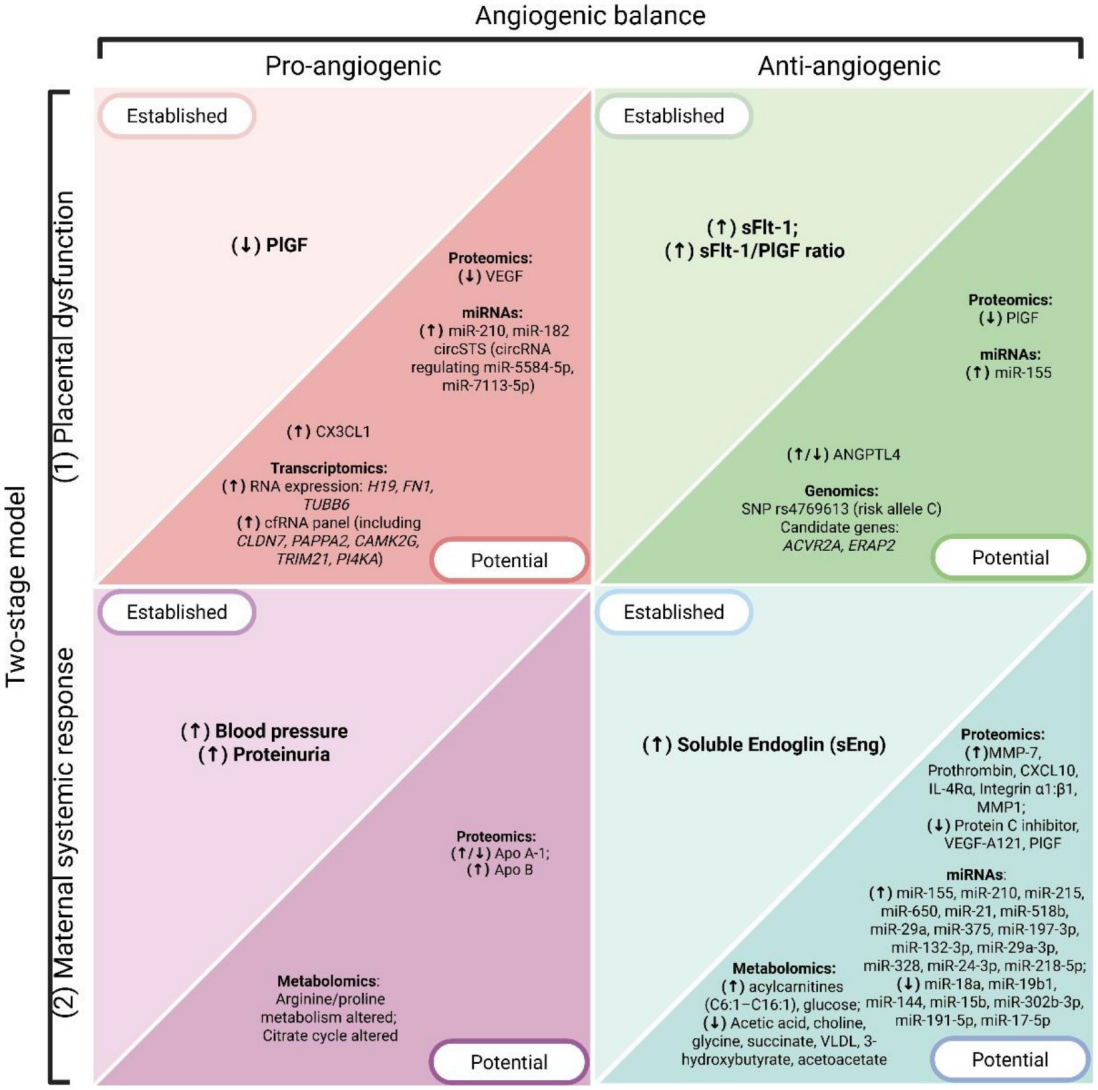
Overview of PE hallmarks and associated biomarkers, organized according to the two-stage model (Stage 1: placental dysfunction; Stage 2: maternal systemic response) and the angiogenic balance (pro-angiogenic vs anti-angiogenic factors). Biomarkers are grouped as established (currently used in clinical practice or guideline-endorsed) and potential (emerging candidates identified through omics technologies). Arrows indicate the direction of dysregulation in PE, where (↑) denotes overexpression or increased levels and (↓) denotes downregulation or decreased levels. Created with BioRender.com.

Angiogenesis is regulated by a balance between pro-angiogenic and anti-angiogenic factors. Within the framework of the two-stage model, these alterations emerge initially as placental dysfunction (Stage 1) and later manifest as a systemic maternal response (Stage 2), with angiogenic imbalance as a central hallmark of the disease (Figures 2, 3). Vascular endothelial growth factor (VEGF) and placental growth factor (PlGF), produced by the placenta, are fundamental for vasculogenesis, angiogenesis, and placental development (29). One of the first angiogenic biomarkers studied in PE was VEGF in 1995 (30). VEGF, a specific factor for endothelial cells, induces endothelial proliferation and chemotaxis and stimulates the formation of new blood vessels. Additionally, VEGF increases microvascular permeability and promotes coagulation, distinctive characteristics of PE, making it a good candidate as an angiogenic biomarker in PE (29–31).

However, different studies have shown contradictory results regarding VEGF levels in women with PE. While several studies demonstrated that circulating concentrations of VEGF are elevated in these women, recent studies have shown that free VEGF levels in serum decrease in PE patients. These discrepancies are attributed to the limitations of conventional methods, such as sandwich-type Enzyme-Linked Immunosorbent Assay (ELISA), which struggle to measure VEGF protein complexes accurately. Consequently, VEGF has been deemed an unreliable biomarker for PE, prompting researchers to focus on other angiogenic and antiangiogenic factors and to explore new methods for effective measurement (31).

In 2003, two independent groups, Koga et al. (32) and Levine et al. (33), identified the importance of anti-angiogenic factors by evaluating the balance between VEGF and its natural antagonist, sVEGFR-1 (soluble vascular endothelial growth factor receptor-1) or sFlt-1 (soluble fms-like tyrosine kinase-1). They reported an increase in circulating levels of sFlt-1 in patients with PE compared to controls without PE (32). This increase was also associated with a reduction in the expression of PlGF and VEGF (33).

In 2004, *in vitro* experiments showed that normal villous explants induced endothelial cell migration and tube formation *in vitro*, both attenuated by preincubation with exogenous sFlt-1. The removal of sFlt-1 by immunoprecipitation significantly restored migration and tube formation to levels comparable to those induced by normal conditioned medium, demonstrating that elevated levels of sFlt-1 in PE are responsible for inhibiting angiogenesis due to the dysregulation of the VEGF/PlGF axis (34).

Between 2004 and 2005, Levine et al. identified that high levels of sFlt-1 and reduced PlGF are detectable even up to 5 weeks before the appearance of the classic clinical manifestations of PE, such as hypertension, proteinuria, and glomerular endotheliosis. This suggests that the relationship between sFlt-1 and PlGF could be used for the prediction or diagnosis of PE (33).

A year later, the relationship between sFlt-1 and PE was further strengthened through studies in murine models, demonstrating an imbalance in the production of anti-angiogenic and pro-angiogenic factors. The exogenous administration of sFlt-1 in mice induced a syndrome similar to human PE, including hypertension, proteinuria, and glomerular endotheliosis, directly linking sFlt-1 to the etiopathogenesis of PE (35). This discovery positioned sFlt-1 as a crucial factor and prompted multiple studies focused on its potential for the prediction and diagnosis of PE.

Deficient spiral artery remodeling, a hallmark of Stage 1 placental dysfunction, causes placental hypoxia and ischemia, increasing anti-angiogenic factors like sFlt-1. Hypoxia-inducible factor-1α (HIF-1α) upregulates sFlt-1, exacerbating endothelial damage, while also inducing angiopoietin-like protein-4 (ANGPTL4). Recently, ANGPTL4 has gained attention as a potential PE biomarker due to its proangiogenic effects, modulation of inflammation, and regulation of metabolism (36–39).

ANGPTL4 was first linked to PE by Liu et al. (40), who reported reduced placental and serum ANGPTL4 in early-onset severe PE, impairing trophoblast invasion and angiogenesis due to decreased PPARγ (Peroxisome Proliferator-Activated Receptor Gamma) activation. Conversely, Açikgözoglu et al. (41) observed elevated maternal serum ANGPTL4 in PE and gestational hypertension (GH), suggesting its utility as an early biomarker since levels increased before proteinuria onset. Also, Shu et al. (42) linked ANGPTL4 downregulation by lncRNA (Long Non-Coding RNA) HIF1A-AS2 (Hypoxia-Inducible Factor 1 Alpha Antisense RNA 2) to oxidative stress, apoptosis, and angiogenic dysfunction in PE (42).

Discrepancies in ANGPTL4 findings complicate its use as a biomarker. Elevated serum ANGPTL4 in early PE may indicate a compensatory response (41), while reduced placental ANGPTL4 in advanced stages may reflect hypoxia and reduced PPARγ activity (40). Similar ANGPTL4 levels in PE and GH further limit its specificity.

Similarly, fractalkine (CX3CL1) has emerged as a marker for early-onset PE and placental dysfunction (43, 44). It exists in membrane-bound and soluble forms, the latter produced by ADAM10 and ADAM17 and detectable in serum (45, 46). Szukiewicz et al. (45), were the first to demonstrate that hypoxia and inflammation stimulate CX3CL1 production and release into circulation. Later, Siwetz et al. (46), linked elevated serum fractalkine in severe early-onset PE to TNF-α (Tumor Necrosis Factor Alpha) activation via the NF-κB (Nuclear Transcription Factor Kappa Beta) pathway, highlighting its role in systemic inflammation. Pala et al. (43), found that while serum fractalkine levels showed no significant differences between controls and intrauterine growth restriction (IUGR) groups, amniotic fluid levels were markedly elevated in IUGR, reflecting localized placental hypoxia Variability in CX3CL1 expression between serum and amniotic fluid, along with small cohorts and inconsistent assays, limits its clinical application, as noted by Szewczyk et al. (47).

While recent biomarkers such as ANGPTL4 and CX3CL1 show potential, their findings remain inconsistent and insufficient for clinical implementation. These observations reinforce the concept that the interplay between pro- and anti-angiogenic factors is not only central to the two-stage model but also provides a framework for interpreting both established and emerging biomarkers (Figure 3). Larger, standardized studies are crucial to address these discrepancies. Meanwhile, well-established biomarkers like sFlt-1 continue to hold greater promise for clinical application due to their more robust evidence base and validated role in PE prediction and diagnosis.

### 5.1 The “antiangiogenic state” in preeclampsia

sFlt-1 (sVEGFR-1) is a soluble protein derived from an alternative splicing variant of the VEGFR-1 (Flt-1) receptor gene, and its upregulation is one of the best-characterized examples of the anti-angiogenic state that defines Stage 1 placental dysfunction and propagates into Stage 2 maternal systemic response (Figures 2, 3). This transmembrane receptor is crucial for the stabilization of endothelial cells in mature blood vessels, as well as the fenestrated and sinusoidal endothelium of the renal glomerulus, brain, and liver. As a circulating protein containing the same extracellular domain as Flt-1, sFlt-1 competes with Flt-1 for its ligands, particularly PlGF and VEGF, exhibiting strong antagonist activity (Figure 2). Additionally, it can form dominant negative complexes with the mitogenically competent VEGFR-2 (34).

The expression of sFlt-1 is associated with the angiotensin II pathway. The type 1 angiotensin II receptor is the main regulator of vascular tone at the systemic level in adulthood. Its activation triggers signaling pathways that activate the NF-κB, NADPH oxidase, and TNFα, increasing the production of antiangiogenic mediators such as soluble endoglin and sFlt-1. Overactivation of this pathway causes a vasopressor effect and increases peripheral vascular resistance (48).

During the early stages of gestation, there is generally a loss of sensitivity to angiotensin II (48). Levels of sFlt-1 begin to increase in the third trimester and gradually decrease toward the end of pregnancy, which is associated with a reduction in peripheral vascular resistance at the beginning and end of pregnancy. Various proangiogenic factors are expressed in trophoblastic cells and the placenta during gestation, with PlGF being one of the most characterized, predominantly in the syncytiotrophoblast. PlGF, a member of the VEGF family, promotes angiogenesis under conditions of ischemia, inflammation, and wound healing, acting as a VEGF enhancer through its binding to high-affinity Flt-1 receptors on endothelial cell membranes (35, 48). During gestation, the concentration of PlGF increases initially, peaks in mid-pregnancy, and then gradually decreases toward term.

In patients with PE, increased signaling of the type 1 angiotensin II receptor has been evidenced from the beginning of gestation (48), which prematurely and sustainedly elevates the concentrations of sFlt-1 secreted by the placenta throughout pregnancy. This causes an early decrease in circulating levels of PlGF, resulting in an antiangiogenic state that causes abnormal placentation due to insufficient remodeling of maternal spiral arteries and consequent placental ischemia (35). The increase in sFlt-1 in PE also antagonizes VEGF signaling, preventing its interaction with Flt-1. As a result, endothelial dysfunction occurs, decreasing the production of prostacyclins and nitric oxide and releasing procoagulant proteins, aggravating placental and maternal endothelial dysfunction, as well as the clinical signs of PE, such as hypertension and proteinuria (35).

While there is a substantial amount of evidence supporting the relationship between sFlt-1 and PE, the elevation of sFlt-1 is not evident in all cases of PE. Different studies have shown that women with severe LO-PE or term PE may present circulating sFlt-1 values within a normal range, suggesting that other antiangiogenic factors may be involved in the pathogenesis of PE (49).

Endoglin (ENG) is a transmembrane co-receptor of transforming growth factor β (TGF-β) with angiogenic effects, which is involved in various angiogenesis-dependent diseases and associated with endothelial dysfunction, such as PE (50). ENG is predominantly expressed in endothelial cells but also in tissues experiencing active angiogenesis, as well as in non-endothelial histotypes such as vascular smooth muscle cells, fibroblasts, hematopoietic progenitor cells, mesangial cells, and syncytiotrophoblast cells (51).

ENG expression is increased in the placentas of women with PE through mechanisms primarily induced by hypoxia, oxidative stress, and oxysterol-mediated liver X receptor (LXR) activation (50). Overexpression of endoglin results in an increase in its soluble form in the maternal circulation. This soluble isoform, known as soluble endoglin (sENG), has an antiangiogenic effect, unlike the transmembrane form, as described and associated with PE in 2006 by Venkatesha and colleagues (52).

sENG represents the extracellular domain of membrane endoglin, cleaved by the action of metalloproteinases, predominantly matrix metalloproteinase-14 (MMP-14) (50, 53). The antiangiogenic and prohypertensive activity of sENG is due to its ability to bind circulating TGF-β molecules (Figure 2). This way, it interferes with TGF-β1 and ALK1 (activin receptor-like kinase 1) receptor signaling pathways, inhibiting endothelial nitric oxide synthase activation, which disorganizes angiogenesis and promotes vasoconstriction (50). The concentration of sENG in maternal serum begins to increase between 8 and 10 weeks before the appearance of clinical manifestations of PE (54). It has even been observed that sENG concentrations can increase in the second trimester of pregnancy before clinical symptoms of PE (55). Due to these properties, sENG actively contributes to the poor placentation observed in PE, as well as to the pathogenesis and manifestation of its clinical signs and symptoms, especially hypertension and proteinuria.

sENG and sFlt-1 may exhibit synergistic effects in the development of PE (Figure 2). Each operates through separate mechanisms, but as demonstrated in studies with pregnant murine models, the endothelial dysfunction and severe PE phenotype caused by sENG are amplified by the co-administration of sFlt-1 (52, 56).

Thus, it is currently recognized that the combination of proangiogenic and antiangiogenic factors, particularly the balance among PlGF, sFLT1, and endoglin, characterizes the development of PE. Unfortunately, no single test has yet met the required specificity and sensitivity to allow its routine use as a diagnostic or predictive tool in clinical practice (57). Therefore, tests that evaluate the combined measure of these factors have shown promising sensitivity and specificity for the early prediction of PE (55, 58).

## 6 Established and potential molecular biomarkers in preeclampsia

The multifactorial nature of PE presents a significant challenge to biomarker discovery. Traditional biomarkers have been derived from known pathophysiological abnormalities, particularly those involving angiogenic imbalance as a central hallmark of the disease, consistent with the two-stage model (Figures 2, 3).

The NIH defines a biomarker as “a characteristic that is objectively measured and evaluated as an indicator of normal biological processes, pathogenic processes, or pharmacological responses to a therapeutic intervention” (59). An ideal biomarker possesses high specificity and sensitivity, is detectable through minimally invasive sampling procedures, and its concentration should indicate a disease state (60–62). Biomarkers can be categorized according to their clinical applications, with the most recognized being diagnostic, predictive, and prognostic biomarkers (26, 63, 64). Additionally, susceptibility/risk, monitoring, pharmacodynamic, and safety biomarkers have been described (64).

The multifactorial nature of PE presents a significant challenge to biomarker discovery. Traditional biomarkers have been derived from known pathophysiological abnormalities associated with PE through a hypothesis-driven approach (57). Despite this, the discovery of new potential biomarkers remains crucial for a deeper understanding of the mechanisms underlying the pathophysiology of PE. Identifying new biomarkers not only offers insights into the disease mechanisms but also provides potential diagnostic tests that can enable effective therapy. This is particularly relevant for PE, where early detection and intervention can significantly impact maternal and fetal outcomes.

In the second part of this review, we will examine, following a historical approach, the current advances in the search for diagnostic and predictive biomarkers based on uteroplacental dysfunction, referred to here as “Established Angiogenic Biomarkers” that emerged in the Contemporary Age of Medicine. Additionally, we will review potential new biomarkers for the prediction and diagnosis of PE using omics technologies, designated as “Potential Molecular Biomarkers” that emerged in the Postgenomic Era. Figure 1 depicts the milestones, main studies, and findings in these two scenarios.

### 6.1 Techniques for established angiogenic biomarker detection

#### 6.1.1 Conventional methods

The serum measurement of sFLT1, sVEGF, PlGF, and sENG, molecules related to the pathogenesis of PE, was initially performed using quantitative sandwich-type ELISA starting in 2003 (33, 35, 65). However, this method is inefficient for evaluating protein complexes and thus ineffective for assessing the total (free and bound) circulating levels of potential markers (31). Consequently, ELISA kits are restricted to research use only, and their cutoff values are not recommended for clinical use (35). Despite this limitation, many research groups continue to use ELISA measurements due to their cost-effectiveness. It is worth noting that ELISA measurements are recommended only for research purposes and for evaluating single biomarkers.

To address this limitation, clinical-use kits based on immunochemiluminescent assays, like the Elecsys types, have replaced ELISAs as the standard for measuring plasma levels of PE biomarkers. These assays show high correlation with ELISA determinations. Additionally, being automated, they can deliver results in ~18 min, significantly reducing diagnosis time (66). For clinical diagnosis and combined biomarker measurements, such as the sFlt-1/PlGF ratio, it is recommended to use Elecsys sFlt-1 and Elecsys PlGF assays, as their cutoff points are better standardized (66).

#### 6.1.2 Emerging and point-of-care platforms

Beyond conventional ELISA and automated electrochemiluminescent immunoassays, several complementary and emerging techniques have been explored to broaden the methodological landscape. Multiplex immunoassays, such as bead-based Luminex platforms, have enabled the simultaneous measurement of panels including sFlt-1, PlGF, and soluble endoglin from minimal sample volumes, offering efficiency and the possibility of multimarker profiling (67, 68). Similarly, mass spectrometry–based proteomics, initially developed for discovery research, has been adapted for targeted validation of angiogenic proteins with high analytical specificity, thereby bridging experimental findings with potential clinical translation (69).

At the clinical interface, point-of-care strategies have begun to emerge, particularly lateral flow and fluorescence immunoassays for PlGF and sFlt-1/PlGF ratios (70). These rapid, portable assays offer immediate risk stratification and have already been piloted within national care pathways for suspected preterm preeclampsia, supporting timely triage in busy obstetric settings. In parallel, microfluidic “lab-on-a-chip” platforms and biosensors using electrochemical or nanomaterial-based detection are under development, showing clinically relevant sensitivity ranges for angiogenic biomarkers in proof-of-concept studies (71).

Despite their promise, most of these techniques remain costly, technically demanding, or still in early phases of validation, which limits their uptake compared with conventional ELISA and automated immunoassays. These barriers are particularly pronounced in low- and middle-income countries, where limited infrastructure and resource constraints hinder deployment and risk widening inequities in access to timely diagnosis and care (72, 73). The “last mile” conditions in rural clinics often degrade performance unless devices are ruggedized and simplified (74); indeed, many promising point-of-care innovations stall precisely because they are not engineered or validated under the realities of LMIC health systems (72). This underscores the need for harmonization, external validation in large and diverse cohorts, and pragmatic assessments of cost-effectiveness before these approaches can complement or replace conventional methods in routine preeclampsia diagnosis.

### 6.2 Predictive and diagnostic applications of pro and anti-angiogenic biomarkers in preeclampsia

One of the first studies to evaluate multiple serum biomarkers of angiogenesis was conducted by Kusanovic et al. (162). Within the framework of the two-stage model, these studies provided evidence that alterations in the angiogenic balance are detectable before the maternal systemic response becomes clinically apparent (Figures 2, 3). This cohort study included 1622 patients with singleton pregnancies, using the ELISA method to measure sFlt-1, PlGF, and sENG in maternal plasma to evaluate the independent and combined values of these biomarkers in early and late pregnancy stages. They observed that women with EO-PE had lower PlGF concentrations in the first trimester compared to women without PE. In the same study, it was identified that a lower PlGF/sENG ratio (lower circulating PlGF or higher sENG) is associated with PE, and this ratio has better predictive value, with a sensitivity of 100% and specificity of 98% for EO-PE prediction (37). However, other authors have shown that PlGF levels alone are sufficient as a predictor of EO-PE (75).

From these observations, the Fetal Medicine Foundation (FMF) developed a first-trimester screening model, endorsed by the International Federation of Gynecology and Obstetrics (FIGO), which estimates the individual risk of developing PE. The model combines maternal clinical factors, mean arterial pressure (MAP), uterine artery pulsatility index (UtA-PI), and serum measurement of PlGF through ELISA, also known as the triple test. This model provides a risk score ranging from 1 to 100, indicating lower to higher risk, with detection rates of 90%, 75%, and 41% for extremely preterm PE (before 32 weeks), preterm, and term PE, respectively (76). This model captures placental dysfunction in Stage 1 through the measurement of angiogenic factors, even before the onset of maternal systemic manifestations in Stage 2.

Subsequently, the measurement of circulating sFlt-1 was included in this model. This modified model was applied to 57 (0.6%) cases of EO-PE and 246 (2.6%) cases of LO-PE, finding a detection rate of 91.2% and 76.4%, respectively. Preventive interventions with acetylsalicylic acid reduce the risk of early PE by 62% using this model (77).

Other proposed combined markers, such as the sFlt-1/PlGF ratio, have been applied for diagnostic purposes. The sFlt-1/PlGF ratio has shown superior diagnostic power for both EO-PE and LO-PE compared to individual measurements of sFlt-1 or PlGF. For EO-PE, the sensitivity and specificity were even higher, reaching 100% and 95%, respectively (66). Other studies have established that an sFlt-1/PlGF ratio of 85 allows detection of PE before 34 0/7 weeks of gestation, with a sensitivity of 89% and specificity of 97%. It has also been identified that a value of 33 above 20 weeks and 110 at 34 weeks or more allows detection of PE with similar sensitivity of 88% and specificity of 99.5% (78).

The PROGNOSIS study, a prospective multicenter study that included 1,273 patients between 24 0/7 and 36 6/7 weeks of gestation, demonstrated that an sFlt-1/PLGF ratio of 38 or higher is indicative of the development of PE in the next 4 weeks, with a positive predictive value of 36.7%. Likewise, if the ratio is lower than this value, it allows the exclusion of PE within a week (79). These findings exemplify how quantifying the imbalance between pro-angiogenic and anti-angiogenic factors can guide clinical decision-making and risk stratification in PE (Figure 3). All this has led to the use of these biomarkers being part of routine obstetric clinical practice in some European countries (76).

The use of these combined biomarkers has been explored not only for the prediction and diagnosis of the disease but also for predicting adverse maternal and fetal outcomes, as well as determining the appropriate timing for ending the pregnancy in cases of PE. Rana et al. (80) was the first to evaluate the efficacy of the sFlt-1/PlGF ratio focused on predicting complications associated with PE. In 616 women with a high clinical suspicion of PE, they observed an inversely proportional relationship between the severity of the imbalance between pro-angiogenic and antiangiogenic factors through the sFlt-1/PlGF ratio and the remaining duration of the pregnancy (80).

The recognition of the role of angiogenic factors in the pathogenesis of PE and their value as biomarkers has led to the development of precision medicine strategies based on these biomarkers (Figure 3). Thadhani et al. (81) proposed different mechanisms to antagonize the effects of sFlt-1, such as saturating the system with its ligands VEGF and PlGF, administering anti-sFlt-1 antibodies, or using dextran sulfate cellulose columns for apheresis and extracorporeal removal of sFlt-1. In two studies that included patients with preterm PE, this latter procedure produced a reduction in sFlt-1 levels, improvement in proteinuria and blood pressure, without adverse effects for the mother and fetus (81, 82).

It is worth noting that the introduction of angiogenic biomarkers has mainly been valuable for the diagnosis and prediction of EO-PE, while evidence remains limited for LO-PE (83). Nonetheless, as both subtypes share pathogenic mechanisms linked to angiogenic imbalance within the two-stage framework, these targeted therapies may ultimately be relevant to both forms of the disease (Figures 2, 3).

### 6.3 Potential molecular biomarkers in preeclampsia

In the postgenomic era, high-throughput sequencing technologies enabled the detection of various molecular biomarkers for complex diseases like PE (Figure 3). When framed within the two-stage theory—placental dysfunction followed by maternal systemic response—these approaches map how molecular alterations across DNA, RNA, proteins, and metabolites converge on the angiogenic balance that underlies disease progression. These technologies have uncovered molecular signatures in DNA and expression biosignatures in various RNA species, which can serve as diagnostic or predictive biomarkers of PE and reveal underlying molecular mechanisms beyond antiangiogenic factors. Other omics technologies such as proteomics and metabolomics, involving the large-scale study of proteins and metabolites within a biological system, have identified proteins and metabolites that provide insight into PE pathophysiology and can potentially be used as predictive and diagnostic biomarkers of PE. Taken together, these omics approaches highlight a continuum—from genomic predisposition to transcriptional, proteomic, and metabolic dysregulation—that converges on angiogenic and anti-angiogenic imbalance. To maintain coherence with the two-stage model, the following subsections emphasize how omics data inform either Stage 1 placental dysfunction or Stage 2 maternal systemic response (Figure 3).

#### 6.3.1 Genetic and genomic insights into preeclampsia

##### 6.3.1.1 Stage 1: placental dysfunction

PE, as a multifactorial condition, presents severity determined by complex interactions between maternal and fetal genotypes as well as environmental factors (84). The genetic predisposition to PE was first documented in the 1960s when researchers at the Margaret Hague Maternity Hospital in New Jersey observed a higher incidence of PE and eclampsia among sisters, daughters, and granddaughters of affected women compared to daughters-in-law. Subsequently, through the Swedish Birth Register, heritability was estimated at ~55%. Additionally, it was identified that this is also dependent on fetal genetics, establishing that the contributions to PE heritability are of both maternal and fetal origin, with 35% and 20%, respectively (84). These findings have been recently supported by an approach based on Genome-based Restricted Maximum Likelihood (GREML), using chip genotypes from European and Central Asian subjects to estimate PE heritability from single nucleotide polymorphism (SNP). Results estimated that the heritability for maternal PE in Europe is 38.1% (95% CI: 29.3–46.8) and for fetal PE is 21.3% (95% CI: 7.4–35.3). In Central Asia, the heritability found was 54.4% (95% CI: 29.6–79.3) for maternal PE and 42.5% (95% CI: 17.3–67.7) for fetal PE (85), suggesting variability in the genetic component among different populations.

EO-PE, linked to severe placental insufficiency, is suggested to have a stronger genetic component. This form of PE is associated with a higher risk of recurrence within families, indicating significant heritability. In contrast, LO-PE is more related to the maternal body's inability to cope with the increased physiological demands imposed by the growing fetoplacental unit (23). These particularities in the heritability of PE have driven the search for the associated genetic background.

Early studies relied on candidate gene association models, which aim to identify genes by comparing allele frequencies and linkage between cases and controls. From this approach, at least eight different chromosomal regions associated with genes like ACVR2A and ERAP2 that may predispose to PE were identified (84). With the advent of next-generation sequencing (NGS) technologies, genome-wide association studies (GWAS) have overcome some of the inherent limitations of candidate gene studies (86). GWAS are based on the idea that many common diseases are complex and polygenic, with multiple variants, each with minimal but additive effects that can contribute to disease risk (87). These studies use SNPs as units of genetic variation in the genome (88, 89).

One of the pioneering studies and the largest GWAS on PE, which included 4,380 cases and 310,238 controls, was conducted by McGinnis and Steinthorsdottir and collaborators (90). They identified SNPs in linkage within the *FLT1* gene on chromosome 13 (SNP rs4769613, risk allele C, *p* = 3.2 × 10^−8^). The C allele, present in 53% of the studied population, was estimated to confer a risk of PE (OR = 1.22, 95% CI (1.14–1.31) per allele), supporting the relationship of FLT1 with the development of PE. Additionally, two other SNPs of independent association with PE were identified: rs12050029 (A>G) (14% frequency) and rs149427560 (C>T) (6% frequency). SNPs rs4769613 (C>T) and rs12050029 (A>G) were located in placental enhancer regions near *FLT1*, suggesting that these variants may influence *FLT1* expression by affecting gene transcription (90). This suggests that multiple causal variants in the fetal *FLT1* locus contribute to disease risk. Interestingly, there was no difference in PE risk based on parental allele segregation, indicating that the increased risk is mediated by fetal gene expression, possibly in the placenta (90).

SNP rs4769613 C>T has been widely studied due to its high association with PE (84, 91). To understand whether there were effects between this SNP and PE subtype, its association in EO-PE and LO-PE, as well as with pregnancies with small-for-gestational-age (SGA) vs. non-SGA babies, was explored. From this approach, it was identified that SNP rs4769613[C] is more common in LO-PE pregnancies with non-SGA babies (90). These differential effects suggest that genetic variation in the *FLT1* locus—specifically in a placental enhancer region near the gene— not only increases PE risk but also relates to the phenotypic heterogeneity of the disease. However, in other studies evaluating the relationship between fetal genotype rs4769613 (C>T) and *FLT1* and sFlt1 levels in placentas of women with PE vs. control placentas, no association of genotype with protein level has been observed (91, 92).

##### 6.3.1.2 Stage 2: maternal systemic response

In addition to placental pathways, maternal genetic factors play an important role in shaping the systemic response that defines the clinical phase of PE. Early candidate gene studies focused on variants within the renin–angiotensin system (RAS), coagulation factors, oxidative stress pathways, dyslipidemia, and immunoregulatory components, particularly within the HLA region (84). However, these approaches are limited as they start from *a priori* hypotheses of pathogenicity. They have often lacked replication due to issues such as inadequate sample sizes, inaccurate clinical phenotyping, lack of correction for multiple testing, poorly matched control groups, hidden ethnic bias, positive publication bias, and random error (84).

Zhao et al. (93) conducted a pioneering GWAS in the maternal genome, including 1,070 Afro-Caribbean mothers, 723 Hispanic mothers, and 1,257 European mothers from the HAPO study. Significant candidate SNPs were identified in different ethnic groups (Table 2). However, no individual SNP was significantly associated with PE after Bonferroni correction, indicating that findings should be interpreted with caution (94). Other studies, including SNP-based gene set enrichment analyses, have enabled the detection of some molecular pathways related to PE risk. A risk SNP, rs7579169 (T>C), is in the intergenic region near the inhibin beta B (*INHBB*) gene. Other SNPs associated with PE, such as rs12711941 (T>G), rs7576192 (A>G), and rs2681472 (A>G), were also identified near the inhibin gene (95). Inhibin is a glycoprotein that reduces levels of follicle-stimulating hormone (FSH). In pregnancies with PE, inhibin protein levels are much higher compared to normal pregnancies, although this relationship is not yet fully understood (94, 95).

**Table 2:** GWAS-identified genes associated with preeclampsia.

**Phenotype**	**Variant (SNP, CNV)**	**Minor allele**	**Gene**	**Sample origin**	**Sample (case/control)**	**Odds ratio (95% CI)**	**P value**	**Cohort**	**Ancestry**	**Effect**	**Ref**.
PE-HTN	rs17367504	G	*MTHFR*	Mother	1,006/816	0.65 (0.53–0.80)	3.52 × 10^−5^	HUNT	Caucasic (Norwegian)	Protective	(94, 96)
PE	rs17249754	A	*ATP2B1*	Mother	1,006/816	0.8 (0.66–0.97)	0.022			Protective	
PE	rs1693812	G	*PAX5*	Mother	1,006/816	0.85 (0.74–0.99)	0.04			Protective	
PE	rs12964645	T	*PLCD3*	Mother	1,006/816	0.85 (0.73–0.80)	0.046			Protective	
PE	rs7579169	C	*INHBB*	Mother	538/540	1.57 (1.32–1.87)	3.58 × 10^−7^	Australian	Caucasic (Australian)	Risk	(95)
PE	rs1426409	A	*KIAA1329*	Mother	177/115	NR	2.58 × 10^−2^	SOPHIA	Caucasic (Iowa)	No surpassed the Bonferroni-correction.	(93)
PE	rs17686866	A	*ESRRG*	Mother	177/115	0.36 (0.23–0.56)	8.99 × 10^−6^				
PE	rs9831647	A	*LMCD1*	Mother	177/115	NR	1.65 × 10^−2^				
PE	rs10743565	A	*IFLTD1*	Mother	177/115	0.41 (0.27–0.60)	6.77 × 10^−6^				
PE	rs11617740	A	*FGF14*	Mother	21/1,010	16.96 (5.53–51.97)	7.32 × 10^−7^	HAPO	Afro-Caribbean	No surpassed the Bonferroni-correction.	(93)
PE	rs2839440	T	*C21orf121*	Mother	21/1,010	5.31 (2.70–10.42)	1.23 × 10^−6^				
PE	rs12641856	A	*MC4C5B20*	Mother	21/1,010	15.19 (4.98–46.35)	1.74 × 10^−6^				
PE	rs4815879	A	*MCM8*	Mother	21/1,010	14.57 (4.81–44.17)	2.20 × 10^−6^				
PE	rs1248993	G	*BAMBI*	Mother	21/1,010	4.27 (2.28–8.01)	5.95 × 10^−6^				
PE	rs28360974	A	*MUC21*	Mother	21/1,010	10.13 (3.85–26.67)	2.76 × 10^−6^				
PE	rs975369	A	*NPWF*	Mother	21/1,010	4.55 (2.34–8.83)	7.79 × 10^−6^				
PE	rs1556832	T	*ADRA1D*	Mother	21/1,010	4.33 (2.27–8.27)	8.95 × 10^−6^				
PE	rs11600901	A	*SCN2B*	Mother	21/1,010	9.83 (3.58–27.00)	9.23 × 10^−6^				
PE	rs7322722	A	*MYCBP2*	Mother	50/1,201	2.93 (1.90–4.52)	1.23 × 10^−6^		European		
PE	rs10989019	C	*INVS*	Mother	50/1,201	3.21 (1.98–5.20)	2.28 × 10^−6^		European		
PE	rs17412740	A	*LZTS1*	Mother	62/658	6.08 (2.88–12.81)	2.14 × 10^−6^		Hispanic		
PE	rs4769613	C	*FLT1*	Fetal	4,380/310,238	1.22 (1.14–1.31)	3.2 × 10^−8^	GOPEC, ALSPAC, deCODE	Caucasic	Risk	(90)
PE	rs4245909	G	*MECOM*	Mother	PE = 16,743 PE-HT*N =* 15,164 Control = 312.686	0.92 (0.90–0.95)	3.19 × 10^−9^	FINNPEC, FinnGen, Estonian Biobank, and the InterPregGen consortium study	Caucasic	Risk	(96)
PE	rs16998073	T	*FGF5*	Mother	PE = 16,743 PE-HT*N =* 15,164 Control = 312.686	1.12 (1.09–1.15)	1.33 × 10^−9^		Caucasic	Risk	
PE	rs2596471	G	*HLA/ PSORS1C2*	Mother	PE = 16,743 PE-HT*N =* 15,164 Control = 312.686	1.11 (1.07–1.15)	1.98 × 10^−9^		Caucasic	Risk	
PE	rs7862828	C	*AUH/ LINC00484*	Mother	PE = 16,743 PE-HT*N =* 15,164 Control = 312.686	1.1 (1.06–1.13)	1.12 × 10^−8^		Caucasic	Risk	
PE	rs3018700	C	*PGR/ TRPC6*	Mother	PE = 16,743 PE-HT*N =* 15,164 Control = 312.686	1.13 (1.09–1.18)	9.98 × 10^−10^		Caucasic	Risk	
PE	rs10774624	A	*ATXN2/ SH2B3*	Mother	PE = 16,743 PE-HT*N =* 15,164 Control = 312.686	0.92 (0.89–0.94)	2.52 × 10^−10^		Caucasic	Protective	
PE	rs7318880	T	*FLT1*	Mother	PE = 16,743 PE-HT*N =* 15,164 Control = 312.686	1.1 (1.07–1.13)	5.04 × 10^−12^		Caucasic	Risk	
PE	rs1421085	C	*FTO*	Mother	PE = 16,743 PE-HT*N =* 15,164 Control = 312.686	1.1 (1.07–1.13)	1.55 × 10^−11^		Caucasic	Risk	
PE	rs6206744	A	*ZNF831*	Mother	PE = 16,743 PE-HT*N =* 15,164 Control = 312.686	1.1 (1.09–1.17)	9.72 × 10^−11^		Caucasic	Risk	
PE-HTN	rs13306561	G	*MTHFR/ NPPAa*	Mother	PE = 16,743 PE-HT*N =* 15,164 Control = 312.686	0.88 (0.84–0.91)	7.49 × 10^−12^		Caucasic	Protective	
PE-HTN	rs1918969	C	*MECOM*	Mother	PE = 16,743 PE-HT*N =* 15,164 Control = 312.686	0.47 (0.90–0.95)	1.34 × 10^−8^		Caucasic	Protective	
PE-HTN	rs16998073	T	*FGF5*	Mother	PE = 16,743 PE-HT*N =* 15,164 Control = 312.686	1.11 (1.11–1.17)	6.27 × 10^−19^		Caucasic	Risk	
PE-HTN	rs12656497	C	*NPR3*	Mother	PE = 16,743 PE-HT*N =* 15,164 Control = 312.686	1.14 (1.07–1.13)	1.77 × 10^−12^		Caucasic	Risk	
PE-HTN	rs10882398	A	*PLCE1*	Mother	PE = 16,743 PE-HT*N =* 15,164 Control = 312.686	1.11 (1.08–1.14)	1.77 × 10^−13^		Caucasic	Risk	
PE-HTN	rs10843404	C	*PZP*	Mother	PE = 16,743 PE-HT*N =* 15,164 Control = 312.686	1.08 (1.05–1.11)	3.16 × 10^−8^		Caucasic	Risk	
PE-HTN	rs11792858	T	*TSN2*	Mother	PE = 16,743 PE-HT*N =* 15,164 Control = 312.686	1.25 (1.32–1.60)	2.74 × 10^−14^		Caucasic	Risk	
PE-HTN	rs6224	A	*FURIN/ FES*	Mother	PE = 16,743 PE-HT*N =* 15,164 Control = 312.686	1.08 (1.06–1.12)	1.77 × 10^−10^		Caucasic	Risk	
PE-HTN	rs113935429	G	*FTO*	Mother	PE = 16,743 PE-HT*N =* 15,164 Control = 312.686	1.1 (1.07–1.13)	1.07 × 10^−10^		Caucasic	Risk	
PE-HTN	rs167479	T	*RGL3*	Mother	PE = 16,743 PE-HT*N =* 15,164 Control = 312.686	0.9 (0.88–0.93)	6.95 × 10^−13^		Caucasic	Protective	
PE-HTN	rs979791	G	*ACTN4*	Mother	PE = 16,743 PE-HT*N =* 15,164 Control = 312.686	0.92 (0.90–0.95)	0.92 × 10^−9^		Caucasic	Protective	
PE-HTN	rs2208589	G	*PRE × 1*	Mother	PE = 16,743 PE-HT*N =* 15,164 Control = 312.686	1.12 (1.08–1.17)	1.7 × 10^−8^		Caucasic	Risk	
PE-HTN	rs201454025	G	*ZNF831*	Mother	PE = 16,743/ PE-HT*N =* 15,164/ Control = 312.705	1.14 (1.10–1.18)	8.41 × 10^−13^		Caucasic	Risk	

PE, preeclampsia; PE-HTN, preeclampsia and hypertension; NR, no reported.

SNPs associated with protective effects against PE have also been identified, particularly in genes related to the calcium signaling pathway. This pathway includes genes such as *ATP2B, ADRA1D*, and *PLCD3*. Notably, SNP rs17367504 (G>A) has been found in the Norwegian population, and SNP rs13306561 (T>C) has been identified in other Caucasian cohorts (Table 2). These SNPs in the *MTHFR* gene have shown associations with protective effects against PE (94, 96).

Recently, Tyrmi and colleagues conducted one of the largest GWAS on PE, combining data from FINNPEC, the FinnGen project, the Estonian Biobank, and the previously published InterPregGen consortium GWAS. This study included a total of 16,743 women with a history of PE and 15,200 with PE or other maternal hypertension during pregnancy. From this analysis, 19 SNPs associated with PE were found, 13 of which were new. Seven of the novel loci harbor genes previously associated with hypertension traits such as *NPPA, NPR3, PLCE1, TNS2, FURIN, RGL3*, and *PREX1*. Additionally, risk loci were identified near genes involved in placental development (*PGR, TRPC6, ACTN4, PZP*), uterine spiral artery remodeling (*NPPA, NPPB, NPR3, ACTN4*), renal function (*PLCE1, TNS2, ACTN4, TRPC6*), and serum proteostasis maintenance during pregnancy (PZP) (96). This study revealed that genes related to hypertension traits are associated with PE, but many of these genes have additional pleiotropic effects on cardiometabolic, endothelial, and placental function. Moreover, several of the associated loci have no known connection to cardiovascular diseases but harbor genes that contribute to the maintenance of a successful pregnancy, whose dysfunctions can lead to PE-like symptoms.

#### 6.3.2 Predictive utility of polygenic risk scores in preeclampsia

##### 6.3.2.1 Stage 1: placental dysfunction

The diversity of loci associated with PE identified by GWAS allows for the consideration of incorporating polygenic risk scores (PRS) as a potential application of these findings in clinical practice. A PRS is typically derived from GWAS data and calculated as the weighted sum of the effect sizes estimated per SNP. PRS is an estimate of genetic susceptibility to the condition of interest, calculated from the effect size of the risk variants of a trait (97). These approaches have been applied in PE prediction and have shown significant potential for improving the identification of women at risk.

An initial study of PRS application in PE/eclampsia was based on a multi-ethnic meta-analysis that included 20,064 cases with PE and 703,117 controls, and gestational hypertension in 11,027 cases and 412,788 controls. This study identified 18 independent loci associated with these conditions, 12 of which are new. These loci highlight the role of natriuretic peptide signaling (*NPPA, NPR3, FURIN*), angiogenesis (*FLT1*), renal glomerular function (*PLCE1, TNS2, TRPC6*), trophoblast development (*WNT3A*), and immune dysfunction (*MICA, SH2B3*) (98). These findings provide new insights into the underlying mechanisms of PE beyond angiogenesis, weaving relationships with HTN mechanisms.

##### 6.3.2.2 Stage 2: maternal systemic response

PRS derived from these loci effectively predicted PE and gestational hypertension in external cohorts, independently of other risk factors. A strong genetic correlation (rg) was observed between PE/eclampsia and gestational hypertension (rg = 0.71), and between systolic blood pressure (SBP) and gestational hypertension (rg = 0.73) (83). These results suggest that PRS provides an additional tool for PE risk stratification, improving the accuracy of risk prediction algorithms.

Under this approach, Nurkkala et al. (99) in a recent study, demonstrated the relevance of PRS in predicting PE and HDP. This study included 141,298 participants from the FinnGen study, followed from 1969 to 2021, to evaluate the association between Blood Pressure Polygenic Risk Scores (BP-PRS) and PE-specific PRS (PE-PRS) with gestational hypertension and PE. The genetic loci identified in this research include genes related to natriuretic peptide signaling, angiogenesis, renal glomerular function, trophoblast development, and immune regulation.

Additionally, results showed that BP-PRS is strongly associated with gestational hypertension (HR 1.38; 95% CI 1.35–1.41) and PE (HR 1.26; 95% CI 1.23–1.29), capturing the genetic architecture of PE better than PE-PRS alone (99). This way, it is suggested that the inclusion of BP-PRS not only improves the prediction of PE and gestational hypertension but also highlights the strong genetic correlation between hypertension predisposition and the occurrence of hypertensive disorders of pregnancy. This offers new perspectives for early diagnosis and efficient management of these conditions through interventions such as low-dose aspirin administration.

#### 6.3.3 Transcriptomics and mRNA as potential biomarkers in preeclampsia

##### 6.3.3.1 Stage 1: placental dysfunction

Transcriptomics applied to complex diseases such as PE has focused on measuring differential gene expression (DEG). This approach has identified gene sets and candidate pathways, as well as constructed gene networks and disease-specific pathways. Various transcriptomics studies in PE have concentrated on DEG in placental tissue using expression microarray techniques and RNA-seq (100). Except for leptin (LEP) and Flt1, most studies have reported inconsistent lists of differentially expressed genes in PE (101). This inconsistency is possibly due to small sample sizes, variability in matching, different maternal ethnicities, different types of chips and microarray platforms used, and indirect comparison designs (i.e., pooling mRNA from multiple individuals into a single sample) (100, 102). Moreover, many DEG-based studies often require invasive procedures, complicating their clinical application.

In a recent study, Tarca et al. (103) characterized cfRNA transcriptomic changes in maternal whole blood associated with early PE before and at the time of disease diagnosis, revealing a significant increase in *H19, FN1*, and *TUBB6* gene expression in women with early PE at 11–17 weeks of gestation. Notably, both *FN1* and *H19* showed higher expression in placental tissue from women with PE. *H19* was found to reduce cell viability while promoting invasion and autophagy in trophoblastic cells, along with the activation of PI3K/AKT/mTOR pathways. These findings provide direct evidence of transcriptomic alterations that contribute to impaired placentation and abnormal trophoblast function in the first stage of PE.

#### 6.3.4 Stage 2: maternal systemic response

Advances in high-throughput sequencing have overcome some of the limitations of placental DEG studies by enabling the study of cell-free RNA (cfRNA), a fraction of RNA released into the blood from various tissues, through minimally invasive procedures. This has facilitated the application of transcriptomics in the clinical context, enabling the proposal of RNA-based biomarkers for complex diseases such as PE.

Rasmussen et al. (104) analyzed 2,539 plasma samples from 1,840 pregnant women from various cohorts with racial and ethnic diversity. They used cfRNA to track pregnancy progression and predict PE months before clinical presentation. Transcriptomic data were collected and analyzed using a machine learning model based on cfRNA profiles. Samples were collected on average 14.5 weeks before delivery, identifying seven key genes (*CLDN7, PAPPA2, SNORD14A, PLEKHH1, MAGEA10, TLE6, FABP1*) predictive of PE, with a sensitivity of 75% and a positive predictive value of 32.3%. This model identifies women at risk of developing PE from 16 to 27 weeks of gestation, providing a significant window for early interventions and improving obstetric management. These genes are associated with relevant biological processes such as placental angiogenesis, arterial morphogenesis, and embryonic placental development.

The applicability of cfRNA for predicting and diagnosing PE was supported by the work of Moufarrej et al. (105), who used cfRNA measurement in plasma in 404 blood samples from 199 pregnant women at different gestational stages. They identified transcriptomic changes associated with PE, dividing participants into discovery and validation cohorts. Samples collected 12 weeks before, between 13 and 20 weeks, from 23 weeks of gestation, and postpartum were analyzed. From these comparisons, it was demonstrated that changes in cfRNA expression between normotensive and preeclamptic mothers are marked and stable from early pregnancy stages. These changes are enriched in tissue-specific and neuromuscular, endothelial, and immune cell type-specific genes, reflecting key aspects of PE physiology. The identified set of 18 genes includes *CAMK2G, DERA, FAM46A, KIAA1109, LRRC58, MYLIP, NDUFV3, NMRK1, PI4KA, PRTFDC1, PYGO2, RNF149, TFIP11, TRIM21, USB1, YWHAQP5*, and *Y_RNA*. These genes are involved in molecular pathways related to muscle contraction, immune signaling, and endothelial function, highlighting the multifactorial nature of PE.

Based on these findings, the authors developed an 18-gene panel, which could predict PE with a sensitivity of 85% to 100% and a specificity of 85% to 97%. From these results, they suggest creating a liquid biopsy test based on evaluating the expression profiles of these 18 genes, which could predict the risk of PE between 5 and 16 weeks of gestation, well before clinical symptoms appear. Implementing such tests could facilitate the adoption of prophylactic interventions, such as low-dose aspirin use (105).

The integration of transcriptomic biomarkers, particularly cfRNA signatures, provides a bridge between placental dysfunction and the maternal systemic response. While DEG analyses of placental tissue highlight impaired trophoblast and angiogenic pathways consistent with stage 1 of PE, cfRNA studies in maternal circulation reveal systemic endothelial, immune, and neuromuscular alterations characteristic of stage 2. Together, these findings underscore the potential of transcriptomic approaches to capture the continuum of PE pathogenesis and support the development of minimally invasive tools for early prediction and diagnosis. These advancements in molecular biology could revolutionize the management of PE, enable early interventions and significantly improve maternal and neonatal outcomes, although rigorous research and validation studies remain essential to establish their clinical applicability and reliability.

#### 6.3.5 MicroRNAs and RNA species: emerging biomarkers for preeclampsia

##### 6.3.5.1 Stage 1: placental dysfunction

Transcriptomic studies have also enabled the identification of a wide variety of RNA species, such as microRNAs (miRNAs), long non-coding RNAs (lncRNAs), and circular RNAs (circRNAs). miRNAs are related to regulatory processes of the genome and are considered potential biomarkers for complex diseases like PE (Table 3). miRNAs are small RNA species, 22–24 nucleotides long, non-coding, endogenous, and highly conserved. They represent only 2%−3% of the genome but can regulate the expression of ~60% of genes (106). A single miRNA can regulate around 200 different transcripts, affecting various cellular pathways; similarly, the same mRNA can be regulated by multiple miRNAs (107). Different studies report that, during normal pregnancy, the miRNA expression profile changes according to the gestational stage (108, 109). Additionally, ~600 different types of miRNAs have been identified that are synthesized in the placenta (110). Different placental miRNAs have even been described as identified in maternal circulation. Due to their greater stability compared to mRNA, they have been considered possible candidates for biomarkers for pregnancy monitoring (111). The altered expression of some miRNAs in placental tissue and maternal circulation has been associated with various types of pregnancy complications, such as PE, preterm labor, and spontaneous abortion (112).

**Table 3:** Key miRNAs associated with the development of preeclampsia.

**Year published**	**miRNA**	**Effect**	**Target/effect**	**Pathway**	**Tissue/ expression**	**Ref**.
2007	miR-182	↑	*TGFBR2, ↓*,	Proliferation, differentiation, and Trophoblast apoptosis, angiogenesis and inflammation.	Placenta ↑; Plasma -	(109)
			*FOXO1 ↓;*			
			*NDRG1 ↓*.			
2017	miR-223	↓	*STAT3, ↓;*	Apoptosis and response to oxidative stress, complex IL-6/STAT3/miRNA-223 pathway	Placenta ↑; Plasma↑	(116)
			*FOXO1, ↓;*			
			*ICAM1, ↓*.			
2013	miR-29a	↑	*MCL1, ↓*	Trophoblast apoptosis, cell cycle, endothelial cells proliferation, angiogenesis. MiR-29a may behave as a tumor suppressor or promoter.	Placenta -; Plasma↑	(119)
			*HBP1, ↓*			
			*PTEN, ↓*			
2018	miR-210	↑	*EFNA3, ↓;*	Proliferation and invasion of trophoblasts; inhibit trophoblast invasion; endothelial dysfunction	Placenta ↑; Plasma↑	(112)
			*KCMF1, ↓;*			
			*HOXA9, ↓*;			
			*HSD17B1, ↓*.			
2017	miR-126	↓	*SPRED1, ↓*;	Trophoblast angiogenesis, migration, vascular remodeling and angiogenesis,	Placenta -; Plasma -	(116)
			*PIK3R2, ↓*;			
			*CXCL12, ↓*.			
2017	miR-21	↑	*PDCD4, ↓*	Trophoblast cells proliferation, invasion, migration, apoptosis, endothelial dysfunction and angiogenesis.	Placenta -; Plasma↑	(116)
			*PTEN, ↓*			
			*VEGF-A, ↓*.			
2021	miR-650	↑	*ING4, ↓;*	Proliferation, apoptosis, and inflammation.	Placenta ↑; Plasma↑	(117)
			*TP53 ↓*,			
			*VEGF-A ↓*.			
			*PLGF, ↓;*			
			*ITGA5, ↓*.			
2018	miR-221	↑	*c-kit, ↓;*	Proliferation, invasion, migration, angiogenesis, cell cycle progression and increases cell proliferation.	Placenta ↑; Plasma↑	(112)
			*CDKN1B, ↓;*			
			*TIMP3, ↓*.			
2018	miR-376c	↓	*Nodal ↓;*	Trophoblast invasion, Nodal signaling pathways, cell proliferation, differentiation, migration and immune regulation.	Placenta ↑; Plasma↑	(112)
			*TGF-β, ↓*			

↑ Up-regulation; ↓ Downregulation; - unclear evidence about regulation.

The first study that described miRNAs associated with PE was by Pineles et al. (109), in which the miRNAs miR-210 and miR-182 were highly expressed in preeclamptic placentas using RT-PCR. These miRNAs have been widely studied and are highlighted in immunological processes, being important regulators in inflammation, T cell differentiation and function, and NK cell modulation (113, 114). Subsequently, other miRNAs associated with PE were identified, such as miR-155, which is upregulated in the placentas of women with PE and can affect trophoblast migration and differentiation by inhibiting cell proliferation through cyclin D1 suppression (108, 115).

From these findings, different research groups, using various approaches, have identified numerous miRNA candidates and biological pathways related to PE, allowing PE to be recognized beyond the antiangiogenic hypothesis.

#### 6.3.6 Stage 2: maternal systemic response

Among these studies, one by Jairajpuri et al. (116), using expression microarrays, evaluated the differential expression profile of circulating maternal miRNAs concerning disease severity. They identified an increase in the expression of miR-155, miR-210, miR-215, miR-650, and miR-21, and a decrease in miR-18a and miR-19b1. Additionally, they identified four new miRNAs: miR-518b and miR-29a, which were upregulated, and miR-144 and miR-15b, which were downregulated in severe PE compared to mild PE. These results confirm that miRNAs not only contribute to the pathogenesis of PE but can also predict its severity (116).

On the other hand, Akgör et al. (117) used RT-PCR to analyze circulating maternal miRNAs and identified 11 miRNAs related to PE, suggesting their use as potential biomarkers for the non-invasive prediction and diagnosis of the disease. Eight of these miRNAs (miR-210, miR-375, miR-197-3p, miR-132-3p, miR-29a-3p, miR-328, miR-24-3p, and miR-218-5p) were upregulated, while the remaining three (miR-302b-3p, miR-191-5p, and miR-17-5p) were downregulated (117). These results add to the evidence on miR-24 and miR-29a, which are involved in biological processes such as angiogenesis, inflammation, and hypoxia as potential markers for PE (118, 119).

In 2021, a pioneering study provided a comprehensive analysis of the human placental transcriptome using high-coverage RNA-Seq in rRNA-depleted placental samples (~100 million reads in 302 placental samples). Additionally, other RNA species, such as miRNA and circRNA, were characterized. The study found that the placenta contains multiple abundant and common circRNAs. circRNAs, functioning as miRNA sponges, can exert regulatory effects on them. In this study, a new circRNA (circSTS; chrX: 7,514,882–7,516,290) was identified that could modify the function of miR-5584-5p and miR-7113-5p. However, the functional consequences of the miRNA–circRNA interaction remain poorly understood, so these findings should be taken with caution (100).

Integrative analyses of miRNA, circRNA, and DEGs enabled the construction of gene networks and interaction pathways, revealing dysregulated gene sets in PE, particularly those involving the PSG (pregnancy-specific glycoprotein) family. Additionally, other key reproductive importance genes were identified, such as KIR immune receptor genes, which mediate the interaction between natural killer cells in the uterus and trophoblast cells. The study also found that 15% of women with PE and 8% with fetal growth restriction (FGR) had FSTL3 levels higher than 61.4 ng/ml. The study identifies overlapping transcriptomic alterations in PE and FGR, with FSTL3 emerging as a promising biomarker for adverse pregnancy outcomes (100).

Despite the abundant evidence and application in understanding PE through transcriptomic studies, the clinical application of cfRNA and other RNA species in early or mid-pregnancy detection is limited. Particularly, they tend to be disregarded in favor of usual detection methods based on biochemical, biophysical, and ultrasound markers, which may be more cost-effective and efficient (120). The study of the association between miRNAs and PE is relatively recent. However, it is essential to carry out more research that provides an understanding of the cellular miRNA mechanisms involved in developing PE. From the perspective of the two-stage model, altered miRNA and circRNA expression observed in the placenta reflects the first stage of defective placentation, while their detection in maternal blood highlights the second stage, where systemic responses become clinically evident. Together, these findings position non-coding RNA species as promising, though still emerging, biomarkers for both stages of the disease continuum.

#### 6.3.7 Proteomics in preeclampsia biomarker discovery

##### 6.3.7.1 Placental dysfunction

Proteomics studies provide an integrated view of individual disease processes at the protein level through the simultaneous analysis of hundreds of proteins, enabling the comparison of protein patterns between healthy and diseased groups (121). These studies have been primarily conducted using techniques such as Mass Spectrometry (MS) for the qualitative and quantitative analysis of proteins and their post-translational modifications (122). MS-Proteomics techniques have contributed to the identification of new biomarkers of PE in a range of biological specimens, including placental tissue, placental trophoblast cells, and maternal biofluids such as blood serum, plasma, and urine (57). However, biofluids have been prioritized as noninvasive samples, making the discovery of differentially regulated proteins in these tissues more promising for clinical biomarker applications. Table 4 presents a summary of the main findings in proteomic studies of these tissues.

**Table 4:** Proteomic biomarkers linked to preeclampsia.

**Year published**	**Sample type**	**Sample (case/control)**	**EO/LO-PE**	**Main findings and potential protein markers**	**Ref**.
2004	Serum	6/6	EO	↑: clusterin.	(123)
2009	Plasma	23/23	EO/LO	↑: 23 proteins: clusterin, Apo B, insulin-like growth factor binding protein complex acid labile chain, endoglin, and serum amyloid P-component. ↓: 12 proteins: afamin precursor, fibronectin, and plasma protease C1 inhibitor.	(128)
2009	Plasma	6/6	EO	↑: 75 kDa single chain vitronectin. ↓: 65 kDa moiety of the 2 chain vitronectin antichymotrypsin, SERPINA3 and Kininogen 1	(127)
2009	Plasma	39 (appropriate birth weight for gestational age baby (*n =* 27), or a small for gestational age baby (*n =* 12)/57	EO	↑: Fibrinogen a-chain, Clusterin isoform 1, C3, human serum amyloid A4 and a-1-antichymotrypsin. ↓: Gelsolin, C6 and C7	(124)
2010	Serum	11/13	EO	↓: Transthyretin and its modified forms, retinol binding protein 4	(134)
2010	Serum	60 [mild PE (*n =* 30), severe PE (*n =* 30)]/58. 70 later developed mild PE (*n =* 30), severe PE (*n =* 40)/79	EO	Clinical PE: 34 proteins were differentially expressed. ↑: fibronectin, pappalysin-2, choriogonadotropin b, ApoC-III, cystatin-C, vascular endothelial growth factor receptor-1, and endoglin. ↓: Matrix metalloproteinase-9, filamin-A, talin-1. Severe PE Preclinical PE: 38 proteins were differentially expressed ↑: C-reactive protein, tubulin b-1 chain, complement factor H-protein 5 ↓: Fibrinogen b-chain, Plasma protease C1 inhibitor	(130)
2010	Placenta tissue	30/30	EO	2 proteins were differentially expressed ↑: Apolipoprotein A-1. ↓: Tropomyosin-3	(132)
2011	Serum	8/5	EO	↑: 27 proteins were differentially expressed including a-2-HS glycoprotein, insulin-like growth factor binding protein acid labile subunit, and a-1-microglobulin/ bikunin	(129)
2011	Urine	Samples gestational weeks: −12 to 16 (PE *n =* 45, control = 86), −20 (PE *n =* 50, control = 49), −28 (PE *n =* 18, control = 17).	EO	10 biomarkers from specimens obtained at week 28 associated with future PE including ↑: Fibrinogen a chain, collagen a chain and uromodulin fragments.	(125)
2013	Serum	31/31; Training set (21 PE, 21 control); Testing set (8 PE, 8 control)	EO/LO	6 proteins: ↑: fibrinogen a, a-1-antitrypsin, Apo-L1, inter-a-trypsin inhibitor heavy chain H4, kininogen-1, thymosin b-4 protein precursors.	(131)
2013	Serum	32 [24–34 weeks (*n =* 15) > 34 weeks (*n =* 17)]/32 [24–34 weeks (*n =* 16) > 34 weeks (*n =* 16)].	EO/LO	The PE biomarkers were not significantly different between EO and LO gestation in either PE or control sera. ↑: Hemopexin ↓: Apo A-I, Apo C-III, Apo-E, haptoglobin, a-2-macroglobin and retinol binding protein 4	(133)
2017	Plasma	76 cases with LO-PE (48 Mild LO PE; 28 Severe LO PE)/90 controls	LO	↑ MMP-7 either in mild or severe PE and low PlGF LOr in gestation (after 22 weeks) were the strongest predictors for LO-PE. 36 proteins were associated with LO-PE in at least one gestational interval.	(136)
2019	Plasma	33/90	EO	At 16.1–22 weeks: ↑ MMP-7, glycoprotein IIbIIIa complex, Sialic acid binding immunoglobulin-like lectin 6 (siglec-6) and activin-A. ↓ PlGF and VEGF-121. At 28.1–32 weeks: ↑ Activated leukocyte cell adhesion molecule, siglec-6, and VEGF-121.	(135)
2019	Blood	45 studies with proteomic data from PE women. First Trimester: B = 6 Second Trimester: B = 9; AF: 1 Third Trimester: PL: 24; B: 11; U: 4; CSF: 2; AF: 1; UCB: 2; UA: 1	EO/LO	9 proteins were independently evaluated in PE studies as potential biomarkers. — ↑ Hemoglobin Subunit Beta, Ceruloplasmin and Histidine-Rich Glycoprotein in all tissues. ↓ Hemopexin in B, PL, UCB but ↑ in the urine. ↑ Fibrinogen Alpha Chain and Clusterin in B and ↓ in PL; — First trimester: ↑Alpha 1 Antitrypsin, Clusterin, Fibrinogen Gamma Chain, Fibronectin 1, Hemopexin; ↓Fibrinogen Alpha Chain, Fibrinogen Beta Chain. Third Trimester: ↑ Alpha 1 Antitrypsin, Clusterin, Fibrinogen Gamma Chain, Fibronectin 1, Ceruloplasmin, Histidine-Rich Glycoprotein, Hemoglobin Subunit Beta, Fibrinogen Alpha Chain, Fibrinogen Beta Chain; ↓Hemopexin, Serum Albumin, Pregnancy-Zone Protein, Gelsolin. Second Trimester: ↑Fibrinogen Gamma Chain, Fibrinogen Alpha Chain, Fibrinogen Beta Chain. ↓Pregnancy-Zone Protein, Gelsolin.	(126)
2022	Plasma	Stanford cohort: 18/18 Detroit cohort: 76 /90	Stanford cohort: EO/LO Detroit cohort: LO	Stanford cohort: ↑ APOB, SPARCL, SAP, DR6, TXD12, XPNPEP1, IGF-I sR, sLeptin R. — Detroit cohort: ↑ MMP7, RAN, PlGF, GSTP1, CDK8, PPID, BMP10.	(138)
2023	Plasma	109/90	EO/LO	Four molecular clusters with distinct clinical phenotypes were identified: Cluster 1 involved metabolic and prothrombotic changes: ↑ prothrombin, ↓ thrombin-inhibiting plasma serine protease inhibitor (protein C inhibitor); Cluster 2 involved maternal anti-fetal rejection mechanisms: ↑ C-X-C motif chemokine 10 (CXCL10), IL-4Rα ; Cluster 3 involved extracellular matrix regulation: ↑ integrin α1:β1 complex and interstitial collagenase (MMP1); Cluster 4 involved angiogenic imbalance: ↓ PlGF, VEGF-A isoform 121.	(137)

↑ Overexpressed; ↓ Underexpressed; B, blood; PL, placenta; U, urine; CSF, cerebrospinal fluid; AF, amniotic fluid; UCB, umbilical cord blood; UA, umbilical artery; EO, early onset; LO, late onset.

The first report of a proteomic study on PE was published in 2004 by Watanabe et al. They performed two-dimensional electrophoresis (2-DE) on sera from six patients with PE and six normal pregnant women, followed by MALDI-TOF-MS and peptide mass fingerprinting, identifying overexpressed Clusterin in the PE group. This finding was confirmed in sera from an additional 80 preeclamptic women and 80 normal pregnant women using immunoassays, which showed that Clusterin levels were significantly higher in preeclamptic women (123). Clusterin, a secreted heat shock protein or chaperone molecule in response to stress, potentially offers protection against cytotoxic agents (123). It has been proposed that Clusterin overexpression may result from endothelial dysfunction or renal issues related to PE (123). Additionally, Clusterin has been reported as upregulated in several proteomic studies, including both EO-PE and LO-PE (Table 4). These findings suggest that Clusterin could be a potential global biomarker for PE.

Most proteomic studies have focused on the identification of biomarkers in EO-PE, leading to the discovery of various potential biomarkers, primarily in serum and plasma. These biomarkers are associated with processes central to abnormal placental development, such as inflammation, coagulation, angiogenesis, and lipid metabolism. In addition to Clusterin, the most common findings in EO-PE include the overexpression of proteins such as multiple isoforms of fibrinogen, including the fibrinogen alpha chain (124–126), and vitronectin (127). Inflammatory and acute phase proteins such as serum amyloid P-component (128), alpha-2-HS glycoprotein (129), and C-reactive protein (130) have also been consistently overexpressed. Similarly, protease inhibitors like alpha-1-antitrypsin (131) have shown increased levels. Additionally, some studies have found overexpression of apolipoproteins such as Apo B (128) and Apo A-1 (132), although Apo A-1 has also been found to be underexpressed in other studies, along with Apo C-III and Apo-E (133).

Underexpressed proteins found in PE include inflammatory and acute phase proteins such as transthyretin and retinol-binding protein 4 (134), protease inhibitors such as plasma protease C1 inhibitor (128), antichymotrypsin, SERPINA3, and kininogen 1 (127). Additionally, proteins related to the innate immune system, such as gelsolin and complement proteins C6 and C7, have also been underexpressed (124). Proteomic studies have further supported the underexpression of angiogenic factors such as VEGF and PlGF (135), consistent with impaired placental angiogenesis.

#### 6.3.8 Stage 2: maternal systemic response

Although fewer studies have focused specifically on LO-PE, proteomics has identified several biomarkers reflecting systemic vascular, immune, and metabolic responses. The pioneering study by Erez et al. (136) focused on LO-PE and identified the overexpression of MMP-7 in both mild and severe LO-PE, with low PlGF levels being the strongest predictors for LO-PE. Other overexpressed proteins identified in LO-PE include prothrombotic proteins such as prothrombin (137); inflammatory proteins such as C-X-C motif chemokine 10 (CXCL10) and Interleukin-4 Receptor Alpha (IL-4Rα) (137); and proteins related to extracellular matrix regulation, such as the integrin α1:β1 complex and interstitial collagenase (MMP1) (130). Additional proteins such as APOB, SPARCL, SAP, and DR6 have also been reported as overexpressed (138).

Several underexpressed proteins in LO-PE have also been described, including the thrombin-inhibiting plasma serine protease inhibitor (protein C inhibitor) (137). Additionally, underexpressed angiogenic proteins such as PlGF and VEGF-A isoform 121 have been observed in LO-PE, consistent with the antiangiogenic state that characterizes the disease (137). These patterns reflect the systemic endothelial dysfunction, prothrombotic changes, and inflammatory environment that dominate the second stage of PE.

Proteomic studies have contributed significantly by identifying hundreds of proteins that could serve as potential biomarkers in both early and late forms of PE. These studies have also highlighted the complexity of the mechanisms underlying the pathophysiology of PE. However, the biomarker discovery phase should be followed by biomarker verification and validation. Only a small number of the discovered markers have been further validated, such as Clusterin, Fibrinogen, Fibronectin, Angiotensinogen, Complement 4, Hemepexin, and Galectin-3, which have demonstrated clinical utility in predicting and diagnosing PE (57). Therefore, before these proteomic biomarkers can be included as clinical biomarkers, further studies and validations are necessary to ensure their reliability and clinical applicability in PE. From the perspective of the two-stage model, proteomic alterations in EO-PE reflect primary placental dysfunction, while those in LO-PE highlight maternal systemic inflammatory and vascular responses. Together, proteomics bridges both stages of PE pathogenesis and underscores the need for integrated multi-omics validation.

#### 6.3.9 The role of metabolomics in preeclampsia biomarking

##### 6.3.9.1 Stage 1: placental dysfunction

Another promising approach in biomarker discovery is metabolomics, which aims to identify metabolites correlated with diseases and environmental exposures. Metabolites are the substrates and products of metabolism that drive essential cellular functions such as energy production and storage, signal transduction, and apoptosis (139, 140). Metabolomic approaches focus on identifying metabolites and metabolic pathways associated with phenotypes and integrating this knowledge with functional and mechanistic biological studies (141). Given that metabolite biosignatures from human biofluids provide a link between genotype, environment, and phenotype (6), significant efforts have been directed toward identifying these biomarkers for the prediction and diagnosis of PE.

The main methodologies used for metabolite recovery and identification are untargeted (global) (UtM) and targeted mass spectrometry-based metabolomics (TM). UtM measures a broad range of metabolites in a sample without prior knowledge, while TM offers higher sensitivity and selectivity by analyzing metabolites based on prior information, typically used to validate and expand upon UtM results (141). Metabolomic studies aimed at discovering PE biomarkers have analyzed biological specimens like tissues (e.g., placenta) and biofluids. Various methods have been employed to measure potential biomarker molecules, including amino acids, fatty acids, organic acids, and acylcarnitines (142).

One of the first reported MS-UtM studies in PE was in 2004 (143). The study by Jain, Jayasimhulu, and Clark aimed to characterize phospholipids (PHLs) in the placenta of normotensive and PE women. The study found a notable deficiency in plasmenyl phosphatidylethanolamine (PPE), which was associated with oxidative stress as an additional biological factor linked to the pathogenesis of PE. Moreover, an increase in free fatty acids, particularly arachidonic acid, was observed in the PE placentas. This could result from heightened activity of placental phospholipase A2, leading to the production of potent vasoconstrictors such as thromboxanes and isoprostanes, which are elevated in PE.

##### 6.3.9.2 Stage 2: maternal systemic response

In 2007, Turner et al. (144) reported the first MS-UtM study in biofluids, aiming to establish the metabolic profile in plasma of normal and preeclamptic women. The study included 11 normotensive pregnant women and 11 preeclamptic women. The results revealed lower concentrations of lipids such as very-low-density lipoprotein (VLDL) and lower levels of ketone bodies like 3-hydroxybutyrate and acetoacetate in preeclamptic women, suggesting that lipid and ketone body constituents contribute to the discrimination between the two groups (144). These findings support the oxidative stress mechanism as a significant underlying factor in the pathogenesis of PE. Additionally, other metabolites such as glycoprotein, proline, lactate, and various amino acids were present in lower concentrations in the PE group.

In 2008, Kenny et al. (145) conducted a study with an amplified sample that included plasma from 20 preeclamptic women and 20 matched controls. They identified eight metabolites overexpressed in PE: uric acid, 2-oxoglutarate, glutamate, alanine, 2-hydroxy-3-methyl-butanoic acid, 2-ethyl-3-hydroxypropionic acid, xylitol/ribitol, and creatinine, suggesting their potential as biomarkers for PE. Uric acid and 2-oxoglutarate are associated with ischemic conditions and may reflect the oxidative stress and metabolic disruptions characteristic of PE. The findings support the hypothesis that metabolic changes precede clinical symptoms of PE, allowing for early detection and targeted interventions.

In recent years, several metabolomic studies have identified various metabolites associated with PE, both in EO-PE and LO-PE. A recent systematic review by Yao et al. (140) summarized 41 human metabolomics studies conducted from 2000 to 2021, primarily focusing on biofluids to identify potential metabolic biomarkers and pathways linked to PE. The review highlighted frequently upregulated metabolites in EO-PE, including 3-hydroxyisovaleric acid, glucose, propylene glycol, hexanoylcarnitine, octenoylcarnitine, L-octanoylcarnitine, decenoylcarnitine, decanoylcarnitine, dodecenoylcarnitine, lauroylcarnitine, tetradecenoylcarnitine, and hexadecenoylcarnitine. Conversely, metabolites such as acetic acid, choline, formate, glycerol, glycine, L-phenylalanine, and succinate were found to be downregulated.

In LO-PE, commonly upregulated metabolites included glucose, octenoylcarnitine, L-octanoylcarnitine, decenoylcarnitine, decanoylcarnitine, and dodecenoylcarnitine, while downregulated metabolites included linoleylcarnitine, palmitoylcarnitine, oleylcarnitine, and stearoylcarnitine.

Pathway analysis demonstrated the significant involvement of multiple metabolic pathways. Among them, eight were related to amino acid metabolism, including arginine biosynthesis; alanine, aspartate, and glutamate metabolism; arginine and proline metabolism; glycine, serine, and threonine metabolism; valine, leucine, and isoleucine biosynthesis; phenylalanine metabolism; D-glutamine and D-glutamate metabolism; and histidine metabolism. Additionally, three pathways were linked to carbohydrate metabolism: butanoate metabolism; the citrate cycle; and glyoxylate and dicarboxylate metabolism (140). Other affected pathways included aminoacyl-tRNA biosynthesis, associated with translation, and sphingolipid metabolism, related to lipid metabolism (140). These findings indicate that these metabolites and pathways could serve as potential biomarkers for early diagnosis and provide valuable insights into the pathogenesis of PE.

Although metabolomics has highlighted different mechanisms underlying PE pathogenesis, utilizing metabolites as biomarkers presents several challenges. One of the largest challenges is overcoming interindividual metabolite variation, which arises from differences in genetic factors and environmental exposures (139, 140). Additionally, evaluating the biological roles of the identified metabolites is crucial for advancing from biomarkers to understanding disease mechanisms (141). The complexity of metabolic pathways in which each metabolite can be involved complicates the inference of its function and its differences in various contexts. Thus, translating these metabolomics findings into clinical practice must involve comprehensive validation studies, robust statistical analyses to account for individual variations, and integration with other omics data to fully elucidate the biological context and relevance of the identified potential biomarkers in PE (133, 141, 146). From the perspective of the two-stage model, early placental metabolic signatures such as oxidative stress and lipid alterations correspond to Stage 1 dysfunction, whereas systemic metabolic changes in maternal biofluids reflect Stage 2 responses. Together, these findings reinforce the value of metabolomics as a complementary approach to other omics in elucidating PE pathogenesis and developing predictive biomarkers.

## 7 Multi-omics approach in biomarkers research in preeclampsia

Omics technologies applied to biofluids have added additional layers to the biomarker discovery process in PE. Numerous biological markers based on genomics, transcriptomics, proteomics, and metabolomics have helped identify molecular signatures involved in various biological pathways, enhancing our understanding of the underlying pathogenesis of PE. Taken together, these findings converge with the two-stage theory of the disease: genomic and transcriptomic data highlight alterations in placental development, trophoblast invasion, and angiogenic signaling characteristic of the first stage, while proteomic and metabolomic studies often reflect the maternal systemic responses that dominate the second stage, including endothelial dysfunction, inflammation, oxidative stress, and metabolic disturbances. This integrated perspective shows how each omics layer contributes to different points in the disease continuum, supporting theories related to placental perfusion/vascular resistance, endocrine, renal, and immune dysregulation, as well as additional biological processes such as insulin resistance, lipid metabolism disorders, and activated inflammatory responses.

Despite these advances, using these molecular findings as biomarkers remains challenging in efforts to predict PE. Many potential biomarkers have demonstrated poor sensitivity and limited positive predictive values (6).

Consequently, no widely accepted molecular markers have yet proven to be reliable, reproducible, and cost-effective for predicting or diagnosing PE, which obstructs their application in clinical contexts. This underscores the need for continued research and rigorous validation to improve early detection.

Integrative-omics, or multi-omics, approaches that combine two or more omics datasets for data analysis, visualization, and interpretation (141, 146) have been proposed to overcome these limitations. By integrating omics data acquired from the same samples, these approaches can provide a more comprehensive understanding of the molecular landscape of PE and improve the accuracy of diagnosis and prognosis. Recently, such approaches have gained significant interest and are being adopted by research groups worldwide to accelerate progress in understanding complex diseases like PE and in the discovery of clinically relevant biomarkers (133, 147, 148).

## 8 Artificial intelligence in preeclampsia: promises and challenges

Artificial Intelligence (AI) methods, including machine learning (ML) and deep learning (DL), have demonstrated significant potential in maternal and perinatal care. These technologies are particularly valuable for handling complex data to predict and diagnose PE (149). Recently, numerous predictive and classification models have been developed, with most studies focusing on AI-based approaches to predict PE using clinical and paraclinical variables (146, 150, 151).

One of the first and largest clinical studies on PE prediction was conducted in 2019 by Jhee et al. (152). This study aimed to develop ML-based models for predicting late-onset PE using electronic medical records from 11,006 pregnant women in Seoul, South Korea. Maternal clinical and laboratory data from the early second trimester to 34 weeks of gestation were analyzed, including systolic blood pressure, serum creatinine, blood urea nitrogen (BUN), platelet count, urinary protein levels, white blood cell count, and electrolyte levels (e.g., calcium and magnesium). Among the predictive models tested, the stochastic gradient boosting model outperformed others, achieving a C-statistic of 0.924, an accuracy of 97.3%, and a false positive rate of 0.009. These findings demonstrated the potential of ML algorithms to identify meaningful temporal trends, improve prediction accuracy, and surpass traditional statistical approaches.

Later that year, Sandström et al. (153) explored an innovative approach to identify nulliparous women at risk of PE using multivariable predictive models. This study leveraged electronic health data from 62,562 pregnancies and compared three predictive methods: logistic regression with pre-specified variables, backward selection, and a Random Forest model. The models were developed based on early antenatal data, including clinical, social, and medical history, alongside physical measurements such as MAP, proteinuria, hemoglobin, and glucose levels. Among these, the pre-specified logistic regression model performed best, with an AUC of 0.68 for early-onset (< 34 weeks) and preterm PE, outperforming the NICE guidelines at a 10% false positive rate. However, when considering all three models, predictive performance remained modest, with AUCs ranging from 0.58 to 0.68, indicating that none of the approaches surpassed a moderate level of discrimination. Despite progress in personalized risk assessment, the findings underscored the need for external validation and the integration of novel biomarkers to enhance predictive accuracy.

In 2020, Marić et al. (154) developed predictive models for early-onset PE using statistical learning methods. This retrospective cohort study analyzed data from 5,245 pregnancies, of which 561 cases (10.7%) involved PE. Two statistical learning algorithms—elastic net and gradient boosting—were used to construct models based on 67 variables, including maternal characteristics, medical history, vital signs, laboratory results, and medication use. The model for all PE achieved an AUC of 0.79 (95% CI: 0.75–0.83), with a sensitivity of 45.2% and a false-positive rate of 8.1%. For early-onset PE, performance was notably higher, with an AUC of 0.89 (95% CI: 0.84–0.95), a sensitivity of 72.3%, and a false-positive rate of 8.8%. Elastic net proved particularly effective for feature selection, identifying key predictors such as chronic hypertension, a history of preeclampsia, systolic and diastolic blood pressure, and laboratory findings like proteinuria and glucose levels. These results highlighted the utility of statistical learning methods for accurate early prediction of PE.

A recent longitudinal study conducted in China (155), followed 4,644 pregnant women from early pregnancy to develop predictive models for both term (≥37 weeks) and preterm (< 37 weeks) PE. Clinical variables such as maternal age, pre-pregnancy weight, parity, and medical history (e.g., chronic hypertension, diabetes, and systemic lupus erythematosus) were recorded. Biophysical markers, including MAP and UtA-PI, and biochemical markers, such as pregnancy-associated plasma protein A (PAPP-A) and PlGF, were collected during the first trimester. The Voting Classifier achieved the highest performance for predicting preterm PE (AUC: 0.884; sensitivity: 86%; specificity: 83.4%), while Logistic Regression reached an AUC of 0.826 for overall PE prediction, with MAP contributing 44.3% to model accuracy. The inclusion of PlGF and UtA-PI enhanced predictive accuracy for preterm PE. While ML algorithms demonstrated robust performance, their results were comparable to traditional models such as the competing risk model by the Fetal Medicine Foundation (AUC = 0.831 vs. 0.797) (155).

A systematic review by Ranjbar et al. (150), evaluated 128 studies focusing on ML models for PE prediction, integrating maternal characteristics, clinical history, and paraclinical findings. The predictors analyzed included maternal age, body mass index (BMI), serum creatinine, uric acid, platelet count, and ultrasound findings such as uterine artery pulsatility index. The review highlighted nine ML models, with Stochastic Gradient Boosting achieving the highest AUC (0.973) and a false positive rate of 0.009 for late-onset PE prediction. Extreme Gradient Boosting showed strong performance for early-onset PE (AUC: 0.955), while Random Forest demonstrated robust general prediction (AUC: 0.860) (150). These findings emphasize the ability of ML methods to achieve high accuracy with minimal false positives.

Despite their promising results, versatility, and the availability of multimodal and multivariable modeling techniques, these models face significant challenges in clinical generalization. Population variability, inconsistent feature definitions, and limited external validation continue to hinder their broader application. Nevertheless, AI's ability to process extensive and complex datasets offers a valuable opportunity to integrate multi-omics data with clinical and paraclinical variables. This integration presents a promising pathway to address the complexity of PE and improve maternal and fetal care (149).

### 8.1 Artificial intelligence and multi-omics integration

Multi-omics approaches present significant challenges in data processing and interpretation. Consequently, bioinformatics and computational tools are indispensable for minimizing redundancy in these intricate datasets and facilitating the identification of the most relevant metabolites. The rapid expansion of the omics field and digital resources, combined with the vast data and complexity inherent in multi-omics, surpass traditional computational workflows, underscoring the need for innovation. Automated computational workflows markedly expedite data upload and mining processes, offering novel methods for the identification and biological interpretation of data. In this context, AI methods are becoming potent resources for handling multi-omics data (146, 150, 151).

Multi-omics and AI produce complex datasets that necessitate computational tools to identify and correlate omics data across samples, examining their interconnectivity in metabolic pathways relative to the phenotype or pathological processes—patterns that are undetectable by traditional methods. Although AI methods have been applied to PE, particularly utilizing clinical and paraclinical data to efficiently predict PE risk (152, 156–158), the potential of integrating AI methods and multi-omics approaches in PE has not been extensively explored. To our knowledge, the only comprehensive multi-omics study was conducted by Marić et al. (154) in 2022. This prospective study included 33 women in the discovery cohort (17 preeclamptic, 16 normotensive) and 16 women in the validation cohort (12 preeclamptic, 4 normotensive), incorporating transcriptomics, proteomics, metabolomics, lipidomic, and microbiome data from longitudinally collected samples from pregnant women. The study also integrated immune system mass cytometry data for a subset of women and combined it with clinical and demographic data.

For early prediction of preeclampsia within the first 16 weeks of gestation, machine learning models, specifically Elastic Net (EN) regression, were employed to extract the most predictive features from high-dimensional datasets. The urine metabolome model achieved the highest performance (AUC = 0.88), identifying predictive metabolites such as adenine and carnitines, while the proteome model followed closely (AUC = 0.87), with proteins including endothelial growth factor A (VEGFA), leptin (LEP), vascular, L-selectin (SELL), E-selectin (SELE), kinase transmembrane receptor (ROR1), and C-X-C motif chemokine ligand 10 (CXCL10), showing strong predictive value. Validation of the urine metabolite model in an independent cohort yielded an AUC of 0.83. Importantly, integration of multi-omics datasets further improved prediction accuracy (AUC ≈ 0.91). Pathway analyses highlighted tryptophan, caffeine, and arachidonic acid metabolism, while integration with immune cytometry data revealed novel associations between immune and proteomic dynamics (148).

The integration of multi-omics and AI methods holds promise in unraveling the biological mechanisms underlying PE, offering opportunities for biomarker discovery and validation. However, significant challenges remain, the largest being Biomedical Data Inequality (159–161). Recent statistics indicate that over 80% of data from clinical omics studies are derived from individuals of European ancestry, who constitute < 20% of the global population (160). Most PE studies predominantly feature cohorts from Caucasian populations (Table 2). Although AI is revolutionizing biomedical research and healthcare, it also has the potential to exacerbate data inequality, leading to negative impacts (160). Therefore, it is imperative to incentivize omics research with a multiethnic focus and strengthen research efforts in developing countries. This will enable the inclusion of representative cohorts, facilitating the validation and emergence of biomarkers for PE that are truly applicable in clinical contexts worldwide.

## 9 Final remarks and conclusions

Preeclampsia has long been described as a “babel of schemata,” reflecting the persistent difficulty in defining its diagnostic boundaries. The evolution from early clinical observations of hypertension and proteinuria to the use of conventional biomarkers such as proteinuria and blood pressure measurement, and later to angiogenic and anti-angiogenic markers like sFlt-1, PlGF, and soluble endoglin, illustrates how the field has moved from descriptive criteria toward a more mechanistic understanding. Yet, these biomarkers still show limited sensitivity and predictive value, underscoring the need for combined approaches.

The integration of multi-omics approaches with artificial intelligence represents a promising frontier for PE prediction and diagnosis. AI can process complex, multidimensional datasets and enhance risk stratification, paving the way toward precision medicine. However, challenges remain regarding external validation, population variability, and limited generalizability, which currently restrict the clinical adoption of AI-based models.

Beyond these methodological hurdles, the translation of omics technologies themselves into clinical practice remains limited. They demand specialized equipment, trained personnel, and high costs that often confine their use to research settings. These barriers are most evident in low- and middle-income countries, where routine application is impractical and risks widening inequities in maternal health. Simplified essays, cost reduction, and context-specific validation will be essential for equitable implementation in PE.

Therefore, clinical translation remains the greatest challenge. Large, multiethnic validation studies and standardized strategies are urgently required to ensure reproducibility and applicability across diverse populations. Bridging discovery with implementation will be critical to transform biomarker and AI research into effective clinical practice and improve maternal and perinatal outcomes.
